# Human gut bacteria bioaccumulate per- and polyfluoroalkyl substances

**DOI:** 10.1038/s41564-025-02032-5

**Published:** 2025-07-01

**Authors:** Anna E. Lindell, Anne Grießhammer, Lena Michaelis, Dimitrios Papagiannidis, Hannah Ochner, Stephan Kamrad, Rui Guan, Sonja Blasche, Leandro N. Ventimiglia, Bini Ramachandran, Hilal Ozgur, Aleksej Zelezniak, Nonantzin Beristain-Covarrubias, Juan Carlos Yam-Puc, Indra Roux, Leon P. Barron, Alexandra K. Richardson, Maria Guerra Martin, Vladimir Benes, Nobuhiro Morone, James E. D. Thaventhiran, Tanmay A. M. Bharat, Mikhail M. Savitski, Lisa Maier, Kiran R. Patil

**Affiliations:** 1 Medical Research Council Toxicology Unit, University of Cambridge, Cambridge, UK; 2 Interfaculty Institute of Microbiology and Infection Medicine, M3 Research Center, Cluster of Excellence ‘Controlling Microbes to Fight Infection’, University of Tübingen, Tübingen, Germany; 3 European Molecular Biology Laboratory (EMBL), Heidelberg, Germany; 4 Structural Studies Division, MRC Laboratory for Molecular Biology, Cambridge, UK; 5 Randall Centre for Cell and Molecular Biophysics, King’s College London, London, UK; 6 Department of Life Sciences, Chalmers University of Technology, Gothenburg, Sweden; 7 Institute of Biotechnology, Life Sciences Centre, Vilnius University, Vilnius, Lithuania; 8 Medical Research Council Centre for Environment and Health, Environmental Research Group, School of Public Health, Imperial College London, London, UK; 9 Department of Epidemiology and Biostatistics, Imperial College London, London, UK; 10 NIHR Health Protection Research Unit in Environmental Exposures and Health, Imperial College London, London, UK; 11 Department of Biochemistry, University of Cambridge, Cambridge, UK

**Keywords:** Cellular microbiology, Bacteria, Chemical biology, Microbiome

## Abstract

Per- and polyfluoroalkyl substances (PFAS) are persistent pollutants that pose major environmental and health concerns. While few environmental bacteria have been reported to bind PFAS, the interaction of PFAS with human-associated gut bacteria is unclear. Here we report the bioaccumulation of PFAS by 38 gut bacterial strains ranging in concentration from nanomolar to 500 μM. *Bacteroides uniformis* showed notable PFAS accumulation resulting in millimolar intracellular concentrations while retaining growth. In *Escherichia coli*, bioaccumulation increased in the absence of the TolC efflux pump, indicating active transmembrane transport. Cryogenic focused ion beam secondary-ion mass spectrometry confirmed intracellular localization of the PFAS perfluorononanoic acid (PFNA) in *E. coli*. Proteomic and metabolomic analysis of PFNA-treated cells, and the mutations identified following laboratory evolution, support these findings. Finally, mice colonized with human gut bacteria showed higher PFNA levels in excreted faeces than germ-free controls or those colonized with low-bioaccumulating bacteria. Together, our findings uncover the high PFAS bioaccumulation capacity of gut bacteria.

## Abstract (web summary)

Human gut bacteria bioaccumulate per- and polyfluoroalkyl substances (PFAS), commonly known as forever chemicals, in intracellular aggregates. Colonization of gnotobiotic mice with bioaccumulating bacteria increases faecal PFAS excretion.

## Main

Environmental contamination by manufactured chemicals has, by some estimates, exceeded the safe planetary boundary^1,2^. Due to the widespread contamination of water and agricultural systems, a vast number of chemical pollutants make it into food and, hence, into the human body^3–8^. The gut microbiota is particularly susceptible to exposure, and adverse interactions therein could cause systemic effects owing to the critical role of the microbiota in host physiology^9–11^. While the effect of chemical pollutants on bacterial growth is being mapped^12^, the impact of gut bacteria on chemicals remains largely an open question.

Among the chemicals of greatest concern are per- and polyfluoroalkyl substances (PFAS), which are often referred to as ‘forever chemicals’. This group of chemicals includes >4,700 compounds^13^ that are used in a wide range of industrial and consumer products, including firefighting foams, waterproof clothes and nonstick cookware. The widespread use of PFAS owes to their exceptional stability resulting from the strength of C–F bonds^14^, and surfactant-like properties due to the presence of strong hydrophilic and hydrophobic groups. Yet, these same properties have made PFAS a concern for environmental and human health^1,4,6,15–19^. The annual health-related cost of PFAS exposure is estimated to be 50–80 billion Euros across Europe^20^. Studies in Europe and the United States have found high prevalence of PFAS in blood^7,21^. Legislative actions are therefore planned to control PFAS levels in drinking water and to restrict usage^22–25^. However, such efforts are not global and, with long environmental half-lives and no efficient route for their removal, PFAS pose a challenge for human and environmental health. Chemical methods to degrade PFAS have had limited success. Most methods show slow kinetics, require multiple processing steps and focus on non-perfluorinated compounds that have some carbon atoms linked to, for example sulfur^26,27^. For reducing PFAS in the human body, ion-exchange resins have been shown to be effective but also exhibited side effects^28^.

Environmental bacteria, such as *Pseudomonas* sp. strains isolated from PFAS-contaminated sites, have been reported to bioaccumulate a sulfur-containing PFAS (perfluorohexane sulfonate, PFHxS). However, the kinetics was slow and solvent preconditioning was needed to facilitate sequestration^29^. Furthermore, due to the surfactant-like properties of PFAS, the molecules are believed to interact with and accumulate mainly in the lipid bilayers^30–32^. Similarly, the interaction of perfluorooctane sulfonate (PFOS)—an 8-C PFAS with a sulfonic acid functional group—with *Lactobacillus* strains was noted as ‘bio-binding’^33^ suggesting binding to the cell surface. Intracellular localization thus remained an open question. As the gut microbiota is a critical interface between exposure and the human body, we investigated how gut bacteria interact with PFAS.

## Results

### Gut bacteria sequester and transform chemical pollutants

**Fig. 1:**
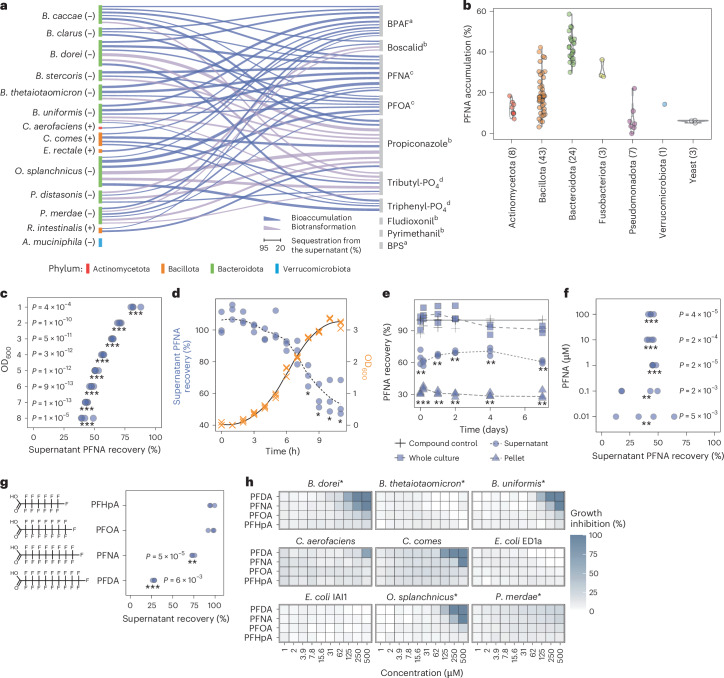
Abundant gut bacterial species bioaccumulate and tolerate PFAS over a broad concentration range. **a**, Specificity of human gut bacteria to sequester (bioaccumulate and biotransform) chemical pollutants during a 24-h growth period as identified using mass spectrometry. Links between bacterial species and pollutant denote >20% depletion. The link thickness is proportional to the median depletion from 6 replicates (3 biological, 2 technical; initial pollutant concentration = 20 μM) (Supplementary Tables 7 and 8). *C. comes*, *Coprococcus comes*; *E. rectale*, *Eubacterium rectale*; *P. merdae, Parabacteroides merdae*; *R. intestinalis*, *Roseburium intestinalis*. Compound class: ^a^bisphenols, ^b^pesticides, ^c^per- and polyfluorinated alkyl substances, ^d^solvent and plasticizer. **b**, PFNA bioaccumulation in 89 strains spanning major bacterial phyla, and three yeasts. OD_600_ = 3.75; initial PFNA concentration = 20 μM (9.3 mg l^−1^); *n* = 3 technical replicates (Supplementary Tables 3 and 9). **c**, PFNA depletion by *B. uniformis* cultures at different OD_600_ values in PBS buffer and PFNA exposure concentration of 20 μM. *P* values are based on two-sided *t*-test; ***P < 0.001; *n* = 4 technical replicates (Supplementary Table 10). **d**, Kinetics of PFNA depletion during *B.* *uniformis* growth starting with low cell density and 20 μM PFNA. *P < 0.05 and >20% PFNA sequestration from the media compared with the compound control. *P* = 0.015 (8 h), 0.005 (9 h), 0.012 (10 h) and 0.014 (11 h); two-sided *t*-test; *n* = 3 biological replicates (Supplementary Tables 11 and 12). **e**, Kinetics of PFNA depletion by *B. uniformis* at high cell density in PBS over 7 days (OD_600_ = 3.75; initial PFNA concentration = 20 μM). Two-sided *t*-test; **P value < 0.01; ***P value < 0.001 (supernatant compared with the compound control; pellet compared with 0); *n* = 3 biological replicates (Supplementary Table 13). Exact *P* values in Supplementary Table 48. **f**, PFNA is significantly bioaccumulated by *B.* *uniformis* grown in mGAM at a range of initial concentrations (initial OD_600_ = 0.05; initial PFNA concentrations = 0.01–100 μM) compared with the compound control. Two-sided *t*-test; *P* value FDR corrected for number of concentrations tested; **adjusted (adj.) *P* value < 0.01; ***adj. *P* value < 0.001; *n* = 4 technical replicates (Supplementary Table 14). **g**, Bioaccumulation of PFAS compounds with varying chain length by *B.* *uniformis* (OD_600_ = 3.75; initial concentration for all compounds = 20 μM (PFHpA, 7,280 µg l^−1^; PFOA, 8,280 µg l^−1^; PFNA, 9,280 µg l^−1^; PFDA, 10,280 µg l^−1^)). Two-sided *t*-test; **P value < 0.01; ***P value < 0.001; *n* = 3 technical replicates (Supplementary Table 15). **h**, Growth sensitivity of gut bacteria to PFAS is independent of bioaccumulation (*n* = 3 technical replicates). Asterisks denote bioaccumulating bacteria (Supplementary Tables 16 and 17).

**Extended Data Fig. 1:**
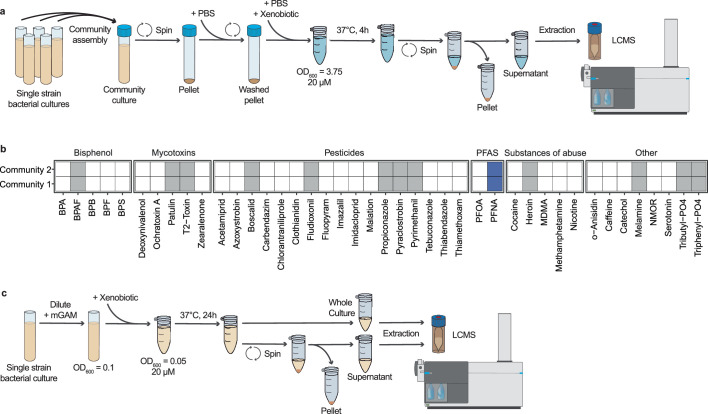
Method setup and results from the community-screen. **a**. Method workflow for the artificial community experiment. Each community consisted of 10 bacteria (community 1: *Bacteroides caccae, Bacteroides dorei, Bacteroides thetaiomicron, Bacteroides uniformis, Bacteroides vulgatus, Colinsella aerofaciens, Coprococcus comes, Eubacterium rectale, Parabacteroides merdae, Roseburia intestinalis;* community 2: *Akkermansia muciniphila, Bacteroides clarus, Bacteroides stercoris, Clostridium difficile, Eggerthella lenta, Eubacterium eligens, Fusobacterium nucleatum subsp*. *animalis, Odoribacter splanchnicus, Parabacteroides distastonis, Ruminococcus bromii*). **b**. 13 out of 42 tested pollutants were sequestered by at least one synthetic gut bacterial community. Coloured squares denote sequestration, that is, >20% reduction from the bacteria-free supernatant. PFAS are marked in blue. n = 6 (3 biological and 2 technical replicates) (Supplementary Table 31). **c**. Method workflow for the single strain experiment.

To assess the potential impact of food-borne pollutants on commensal gut bacteria, we started with a community-based screening approach. We assessed the ability of two mixtures (synthetic communities) of gut bacterial strains to sequester pollutant compounds during 4 h of exposure (Extended Data Fig. 1a). Each synthetic community consisted of 10 human gut bacterial strains (Supplementary Table 1). Allocation of strains to the communities was based on similarity in growth rates. In total, 42 common pollutants were selected for testing against these communities based on reported occurrence in food and for representation of different classes including pesticides, food contact materials and industrial chemicals (Supplementary Table 2). A total of 13 pollutants were found to be depleted by more than 20% by one or both synthetic communities (Extended Data Fig. 1b). Of these compounds, 10 were then tested for depletion by 14 individual strains during a 24-h growth period (Extended Data Fig. 1c). These strains are a subset of the 20 strains forming the two synthetic communities and were selected for their prevalence and abundance in a healthy population, and for phylogenetic and metabolic representation^34,35^ (Supplementary Table 1). In the test with individual strains, 7 pollutants were found to be depleted to more than 20% by at least one of the bacterial strains (Fig. 1a).

By comparing the whole culture and supernatant concentrations to compound controls, we were able to distinguish the observed sequestration between bioaccumulation and biotransformation. Bioaccumulation—storage of the pollutant without modification—was defined as compound sequestration to at least 20% from the supernatant but recovery from the whole culture sample, whereas biotransformation was defined as both supernatant and whole culture sample showing more than 20% depletion.

The pollutants bioaccumulated by gut bacteria included perfluorooctanoic acid (PFOA) and perfluorononanoic acid (PFNA), which belong to the chemical group of PFAS. Of the tested bacterial species, nine—*Bacteroides caccae*, *Bacteroides clarus*, *Bacteroides dorei*, *Bacteroides stercoris*, *Bacteroides* *thetaiotaomicron*, *Bacteroides uniformis*, *Odoribacter splanchnicus*, *Parabacteroides distasonis* and *Parabacteroides* *merdae*—bioaccumulated PFNA and/or PFOA. The degree of bioaccumulation over 24 h for 20 μM (9.3 mg l^−1^) PFNA exposure varied from 25% (*P.* *distasonis*) to 74% (*O. splanchnicus*) and for 20 μM (8.3 mg l^−1^) PFOA from 23% (*P. merdae*) to 58% (*O.* *splanchnicus*). Given the widespread presence of PFAS in the environment, we further investigated PFAS bioaccumulation in gut bacteria to understand its underlying mechanisms.

### PFAS bioaccumulation across gut bacterial strains

**Extended Data Fig. 2:**
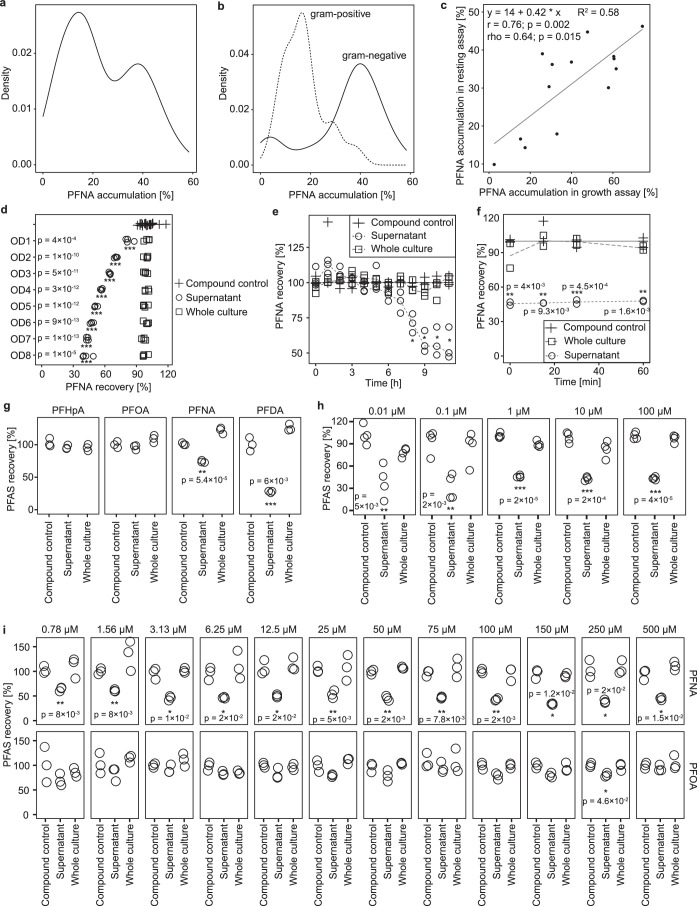
Abundant gut bacterial species bioaccumulate PFAS. **a**. Distribution of PFNA accumulation across all 89 strains (Supplementary Tables 3,9). **b**. Gram-negative strains show on average higher PFNA accumulation compared to gram-positive strains (Supplementary Tables 3,9). **c**. Comparison of PFNA accumulation from the resting assay in PBS compared to the growth assay in mGAM (Fig. 1a) show strong positive correlation (Pearson correlation: r = 0.76, p-value = 0.002; Spearman rank correlation: ⍴ = 0.64, p-value = 0.015). **d**. Data from Fig. 1c including compound control and whole culture samples. PFNA depletion by *B*. *uniformis* cultures of varying OD_600_ in PBS buffer. All ODs show significant PFNA accumulation compared to the compound control. OD_600_ = 1–8; 20 μM initial PFNA concentration; two-sided t-test; *** p < 0.001; n = 4 technical replicates (Supplementary Table 10). **e**. Data from Fig. 1d including compound control and whole culture samples. Kinetics of PFNA depletion during *B*. *uniformis* growth starting with low cell density. A significant amount of PFNA was sequestered from the media after 8 h of growth and onwards. Initial OD_600_ = 0.05; 20 μM initial PFNA concentration; * p<0.05 and >20% PFNA sequestration from the media (Supplementary Tables 11,12). p = 0.015 (8h), 0.005 (9h), 0.012 (10h), and 0.014 (11h) (two-sided t-test; n = 3 biological replicates). **f**. Kinetics of PFNA depletion by *B*. *uniformis* when starting with high cell density in mGAM (OD_600_ = 4). Bioaccumulation of ca. 50% PFNA happens within the time frame of sample collection (ca. 5 min). two-sided t-test; ** p-value < 0.01; *** p-value < 0.001; n = 2 biological replicates (Supplementary Table 32). **g**. Data from Fig. 1g including compound control and whole culture samples. Bioaccumulation of PFAS compounds with varying chain length by *B*. *uniformis*. PFNA and PFDA are significantly accumulated compared to the compound control. OD_600_ = 3.75; 20 μM initial concentration for all compounds; two-sided t-test; ** p-value < 0.01; *** p-value < 0.001; n = 3 technical replicates (Supplementary Table 15). **h**. Data from Fig. 1f including compound control and whole culture samples. PFNA is bioaccumulated by *B*. *uniformis* grown in mGAM at a range of initial concentrations compared to the compound control. initial OD_600_ = 0.05; initial PFNA concentrations = 0.01 to 100 μM; Two-sided t-test; p-value FDR corrected for number of concentrations tested; ** adj. p-value < 0.01; *** adj. p-value < 0.001; n = 4 technical replicates (Supplementary Table 14). **i**. PFNA and PFOA are bioaccumulated by *B.* *uniformis* in PBS at a range of initial concentrations. OD_600_ = 3.75; initial PFAS concentrations = 0.78 to 500 μM; Two-sided t-test; p-value FDR corrected for number of concentrations tested; ** adj. p-value < 0.01; *** adj. p-value < 0.001; n = 3 technical replicates (Supplementary Table 33).

Human gut bacteria are known to harbour extensive intraspecies genomic and functional variability across individuals, including for bioaccumulation of drugs^36^. To assess the differences in intra- and interspecies bioaccumulation capabilities, we tested 89 microbial strains for PFNA sequestration (Supplementary Table 3). We selected PFNA as it is one of the long-chain PFAS that are excreted via faeces and thus are more likely to be encountered by the gut bacteria than short-chain PFAS that are excreted mainly via urine^37^. The 89 strains, which include the 14 strains from the above screen, covered all major bacterial phyla (Fig. 1b) and included 66 human gut commensal strains, 10 probiotic strains, and 10 bacterial and 3 yeast strains isolated from kefir. The 66 gut bacterial strains represent, on average, circa 70% of the abundance of the healthy human gut microbiota^34^. PFNA accumulation showed distinct grouping by phylum, with Bacteroidota showing the highest accumulation (Fig. 1b), and a bimodal distribution that could be divided into two groups using a Gaussian mixture model: low accumulating (51 strains) and high accumulating (38 strains) (Extended Data Fig. 2a). Low-accumulating strains were predominantly Gram positive (43 of 51; 84%), and high-accumulating strains, Gram negative (29 of 38; 76%) (Extended Data Fig. 2b).

Gram-negative bacteria generally have a higher lipid content^38^, which could be a factor underlying PFAS accumulation^31^. However, the divide between the bioaccumulation capacity of Gram-negative bacteria and Gram-positive bacteria is not definitive, with notable exceptions including *Escherichia* *coli*. To further test the influence of lipid content, we tested three yeast species (*Rhodotorula mucilaginosa*, *Candida californica* and *Kluyveromyces marxianus*) that have a high lipid content^39^ comparable to Gram-negative bacteria. The tested yeasts showed only about 5% sequestration (Fig. 1b), suggesting that additional factors beyond lipid content influence bioaccumulation.

### Bioaccumulation features fast sequestration kinetics

To characterize the kinetics of PFAS bioaccumulation, we selected high-accumulating *B. uniformis*, a prevalent gut bacterium^34,35^. PFNA accumulation scaled with cell density, both in non-growing suspension cultures and during bacterial growth (Fig. 1c,d and Extended Data Fig. 2d,e). We next probed the kinetics of PFNA uptake using stationary-phase cultures or cells suspended in phosphate-buffered saline (PBS). In both cases, bioaccumulation occurred within the timescale of sampling (<3 min) (Fig. 1e and Extended Data Fig. 2f). Notably, for *B. uniformis*, no PFNA was released back into the supernatant over the course of 7 days (Fig. 1e).

We next asked whether the fraction of PFAS sequestered was dependent on the exposure concentration. For this, we tested sequestration of PFNA by *B. uniformis* over a broad range of concentrations (0.01–500 μM (4.64 µg l^−1^ to 232 mg l^−1^)). The degree of sequestration remained constant around 50%, both in growing cultures and resting cells (Fig. 1f and Extended Data Fig. 2h,i). Furthermore, the degree of bioaccumulation by *B.* *uniformis* increased with increasing PFAS chain length, from none for perfluoroheptanoic acid (PFHpA, 7C) to 60% in the case of perfluorodecanoic acid (PFDA, 10C) (Fig. 1g and Extended Data Fig. 2g). Despite this high degree of bioaccumulation, *B.* *uniformis* as well as other abundant gut bacterial strains grew well even at high micromolar concentrations (Fig. 1h). Thus, PFAS bioaccumulation occurs within 3 min without inhibiting bacterial growth up to concentrations orders of magnitude higher than known contamination levels of circa 1 nM (refs. ^5,6,15,40^).

### TolC-dependent mechanism of PFAS bioaccumulation in *E. coli*

**Fig. 2:**
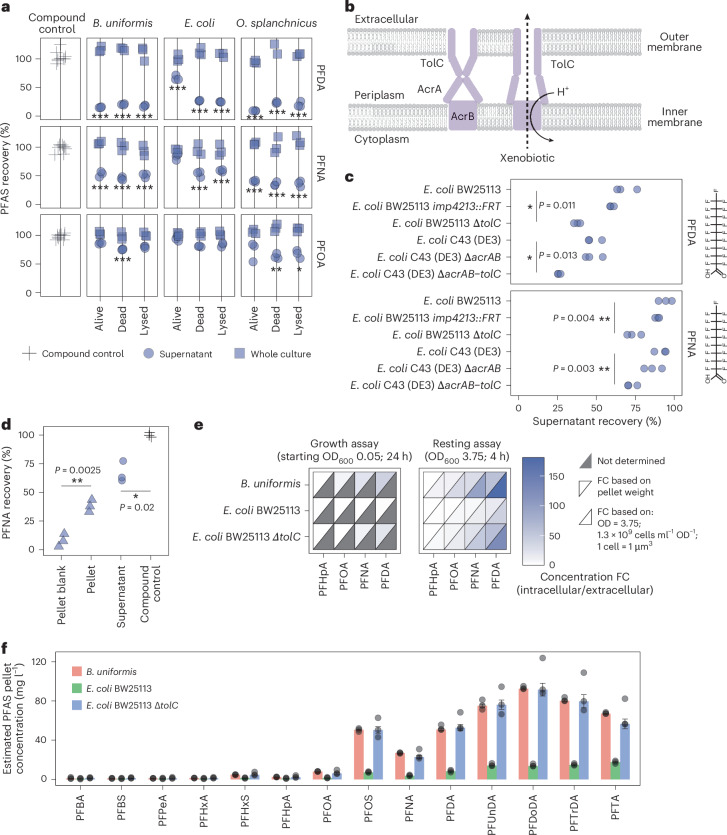
Gut bacteria concentrate diverse PFAS molecules and can export through a TolC-dependent mechanism in *E. coli.* **a**, PFAS bioaccumulation by live, dead (heat inactivated) and lysed (heat inactivated, freeze-thawed and sonicated) *B. uniformis*, *E. coli* and *O. splanchnicus* cultures (OD_600_ = 3.75) in PBS buffer. Two-sided *t*-test; *P* value FDR corrected for number of strains and compounds tested; *adj. *P* value < 0.05; **adj. *P* value < 0.01; ***adj. *P* value < 0.001 and >20% reduction compared with the compound control; *n* = 3 technical replicates (Supplementary Table 18). **b**, AcrAB-TolC efflux pump schematic. The resting state of the pump is depicted on the left. The pump changes conformation to export the xenobiotic (right). TolC can work in combination with other pumps^41,81,82^. **c**, Bioaccumulation of PFDA and PFNA by wild-type *E. coli* strains and corresponding efflux and permeability mutants. Efflux mutants *E. coli* BW25113 ∆*tolC* and *E. coli* C43 (DE3) ∆*acrAB-tolC* showed a ~1.5-fold increase in PFDA and ~5-fold increase in PFNA bioaccumulation. OD_600_ = 3.75; exposure concentration = 20 µM (PFNA, 9.3 mg l^−1^; PFDA, 10.3 mg l^−1^); two-sided *t*-test; *P value < 0.05; **P value < 0.01 compared with the corresponding wild type; *n* = 3 technical replicates (Supplementary Table 19). **d**, PFNA bioaccumulation by *B.* *uniformis* at 0.34 nM (160 ng l^−1^) exposure. Around 37% of PFNA is sequestered from the media into the bacterial pellet. Two-sided *t*-test (supernatant compared with the compound control; pellet compared with the pellet control); *P value < 0.05; **P value < 0.01; *n* = 3 biological replicates (Supplementary Tables 20 and 21). **e**, Capacity of gut bacteria to concentrate PFAS from the media into the bacterial pellet in a growth assay in mGAM or resting assay in PBS at 5 µM PFAS exposure (growth assay: initial OD_600_ = 0.05, 24-h incubation; resting assay: OD_600_ = 3.75, 4-h incubation). *n* = 3 technical replicates (Supplementary Table 22). **f**, PFAS recovery from the bacterial pellets after 1 h of exposure in PBS (OD_600_ = 3.75, PFAS mix of 14 compounds each at a concentration of 1 mg l^−1^). The bars depict the median concentration based on pellet weight; the error bars show standard error; *n* = 3 technical replicates shown as circles (Supplementary Table 23).

**Extended Data Fig. 3:**
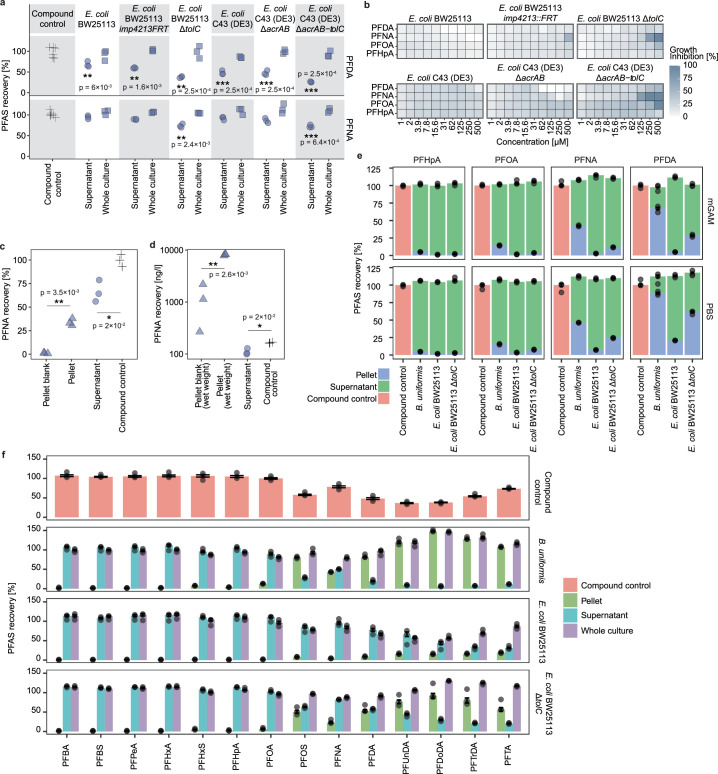
PFAS bioaccumulation in cell pellet. **a**. Data from Fig. 2c including compound control and whole culture samples. Accumulation of PFDA and PFNA by wild-type *E. coli* strains and corresponding efflux mutants. OD_600_ = 3.75; exposure concentration = 20 µM; two-sided t-test; ** p-value < 0.01 and >20% sequestration compared to the compound control; *** p-value < 0.001 and >20% sequestration compared to the compound control; n = 3 technical replicates (Supplementary Table 19). **b**. Efflux mutants show increased PFAS sensitivity. n = 3 technical replicates (Supplementary Tables 16, 17). **c**. Independent measurement of samples from Fig. 2d at Imperial College London supports PFNA accumulation by *B. uniformis* at 160 ng/l exposure concentration. Two-sided t-test (supernatant compared to the compound control; pellet compared to pellet control); * p-value < 0.05; ** p-value < 0.01; n = 3 biological replicates (Supplementary Table 34). **d**. Data from Fig. 2d displayed in a different way: based on wet pellet weight, concentration within the bacterial pellet was a median of circa 8200 ng/l, which is a 50-fold increase compared to the exposure concentration. Two-sided t-test (supernatant compared to the compound control; pellet compared to pellet control); * p-value < 0.05; ** p-value < 0.01; n = 3 biological replicates (Supplementary Tables 20, 21). **e**. Data related to Fig. 2e: PFAS recovery from pellet and supernatant after exposure to 5 µM PFAS in mGAM (initial OD_600_ = 0.05, 24 h incubation) or PBS (OD_600_ = 3.75, 4 h incubation). Bars depict median percent recovery from the bacterial pellet, supernatant or compound control; error bars depict standard error; overlaid points depict single data points; n = 3 technical replicates; (Supplementary Table 35). **f**. Data from Fig. 2f including compound control, whole culture and supernatant samples. PFAS recovery after 1 h exposure in PBS (OD_600_ = 3.75, PFAS mix of 14 compounds each at a concentration of 1 mg/l). For PFAS compounds with a chain length of more than ten carbon atoms, the solubility is limited at this concentration; the low accumulation in *E. coli* BW25113 wild-type pellets acts as a biological control in this case. Bars depict median percent recovery from the compound control, whole culture, supernatant or bacterial pellet; error bars depict standard error; overlaid points depict single data points; n = 3 technical replicates; (Supplementary Table 36).

To investigate how PFAS is bioaccumulated, we first tested whether inactive cell mass (that is, dead, lysed cells) could bioaccumulate PFAS. In resting cell assays, both live and inactivated *B. uniformis* and *O.* *splanchnicus* cells bioaccumulated PFOA, PFNA and PFDA to the same extent (~20%, ~55% and ~85%, respectively). By contrast, live *E. coli* bioaccumulated much lower levels of PFOA, PFNA and PFDA (~5%, ~25% and ~40%, respectively), while inactivated cells bioaccumulated to a similar degree as *B.* *uniformis* and *O.* *splanchnicus* (Fig. 2a). This suggests that PFAS bioaccumulation is not solely a passive phenomenon driven by attachment to membrane lipid bilayers. To test this, we measured bioaccumulation in *E. coli* mutants that lacked one or more genes coding for efflux pump proteins (∆*acrA-acrB*, ∆*tolC*) or altered permeability (*imp4213*) (Fig. 2b). Efflux pumps are a common mechanism used by several bacterial species to reduce intracellular concentration of toxic compounds^41,42^. We reasoned that *E. coli* did not bioaccumulate PFNA to the same extent as other tested Gram-negative bacteria because it could, at least to some extent, pump out the PFAS. As gene deletions can exhibit different phenotypes in different strain backgrounds^43^, we used two *E. coli* strains, viz. BW25113 and C43 (DE3). Mutants lacking TolC showed circa 1.5-fold increase in PFDA and circa 5-fold increase in PFNA bioaccumulation (Fig. 2c and Extended Data Fig. 3a). Consistent with the increased bioaccumulation, these mutants showed increased growth inhibition at higher PFAS exposure levels (Extended Data Fig. 3b). These results show that *E. coli* strains use a TolC-dependent mechanism to limit PFAS bioaccumulation.

### Efficient PFAS bioaccumulation at low exposure levels

We next tested whether bacterial cells could bioaccumulate PFAS at very low exposure levels. *B.* *uniformis* cells were exposed to 0.34 nM (160 ng l^−1^) PFNA, a PFAS concentration observed in water samples across Europe and the United States^15,40^ and below average blood levels^6^. We validated the applicability of our liquid chromatography tandem mass spectrometry (LC–MS/MS) method for low-concentration samples using the US Environmental Protection Agency (US EPA) method^44,45^ and using another, independently developed, LC–MS/MS method (Methods). At this low exposure level, *B.* *uniformis* bioaccumulated 37% of PFNA into the bacterial pellet, concordant with depletion from the supernatant (Fig. 2d and Extended Data Fig. 3c). The estimated concentration within the bacterial pellet (wet biomass) was circa 17.7 nM (8,200 ng l^−1^), a 50-fold increase (Extended Data Fig. 3d). In another experiment with 5 µM (PFHpA, 1.82 mg l^−1^; PFOA, 2.07 mg l^−1^; PFNA, 2.32 mg l^−1^; PFDA, 2.57 mg l^−1^) exposure, the concentration within the wet pellet of *B. uniformis* and *E. coli* BW25113 ∆*tolC* ranged from circa 13 µM (5 mg l^−1^) for PFHpA, 50 µM (21 mg l^−1^) for PFOA, 130 µM (60 mg l^−1^) for PFNA, to 250 µM (129 mg l^−1^) for PFDA, that is, 3-, 8-, 25- and 60-fold increase, respectively (Fig. 2e and Extended Data Fig. 3e). As these estimates are based on wet pellet weight, intracellular concentrations will be higher. Considering the estimated number of cells per optical density (OD) unit (0.6–2 × 10^9^ cells ml^−1^ OD_600_^−1^ (ref. ^38^)) and 1 µm^3^ cell volume^38^, the enrichment of PFAS in bacterial cells would be 1.2–7 times higher than in the wet pellet. These results show the large capacity of bacterial cells to bioaccumulate and concentrate PFAS from their surrounding environment.

Environmental contamination often contains a mixture of multiple PFAS compounds. We therefore investigated the bioaccumulation capacity of bacteria exposed to a mixture of 14 PFAS (C4–C14, and three sulfonated variants), each at a concentration of 1 mg l^−1^ (circa 2 µM for PFNA). Many of these compounds, such as PFNA, PFOA, PFOS, PFDA and perfluoroundecanoic acid (PFUnDA), are among the chemicals of high concern as per the National Report on Human Exposure to Environmental Chemicals of the Centers for Disease Control and Prevention. The results confirm increasing bioaccumulation with increasing chain length (Fig. 2f and Extended Data Fig. 3f). While the *E.* *coli* wild type showed minimal bioaccumulation, *E. coli* ∆*tolC* and *B.* *uniformis* bioaccumulated almost 100% of PFAS with chain length above 10C. The percentage of bioaccumulated PFAS for individual compounds is comparable to a single compound exposure. Thus, at 1 mg l^−1^ per compound, PFAS bioaccumulation is additive across different molecules with minimal mixture effects.

### FIB-SIMS imaging reveals intracellular PFNA aggregates

**Fig. 3:**
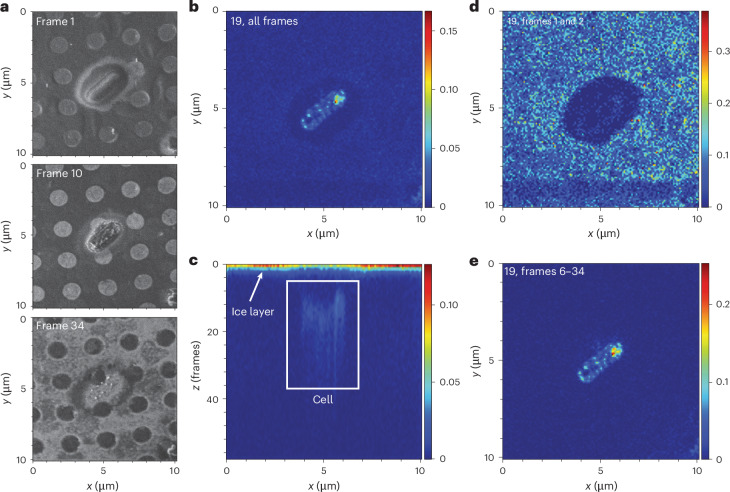
FIB-SIMS imaging shows intracellular bioaccumulation of PFAS by *E. coli ∆tolC.* A given area of the sample is imaged by scanning over it repeatedly with a gallium focused ion beam and analysing the chemical composition of the ablated material using FIB-SIMS. Shown is one of 120 cells imaged (additional images in Extended Data Fig. 6 and Supplementary Data). **a**, Secondary electron images (formed by secondary electrons resulting from the FIB scanning) of three different *Z*-frames of the sample, that is, at three different positions along the *z*-stack, provide spatial images of the imaged cells. The cells are fully embedded in ice in the first panel (frame 1), the second panel (frame 10) shows the interior of the cell and the last panel (frame 34) features the substrate with the cell almost completely removed. **b**–**e**, Top (**b**) and side (**c**) views of the three-dimensional stack of SIMS data for a mass-to-charge ratio of 19, corresponding to fluorine (F^−^), in which the colour scale represents the ion count per extraction. The top view (**b**) shows the lateral distribution (*x*–*y*) of fluorine within the imaged area that is inside the cells. The side view (**c**), corresponding to an *x–z* slice through the stack: for the first few frames, there is a fluorine signal from the whole field of view, stemming from the thin ice layer covering the sample (white arrow in **c**). The fluorine signal away from the cells drops to zero within the first frames. As the cells are initially covered in ice, in the first frames, no highly localized fluorine signal is observed from the cells, as shown by the top view generated from the initial frames (**d**). Once the ion beam mills into the cell, a fluorine signal from the cell can be seen both in the side view (**c**, marked by a white rectangle) and in the top view generated from the corresponding slices (**e**), confirming that the fluorine signal originates from inside the cell.

**Extended Data Fig. 4:**
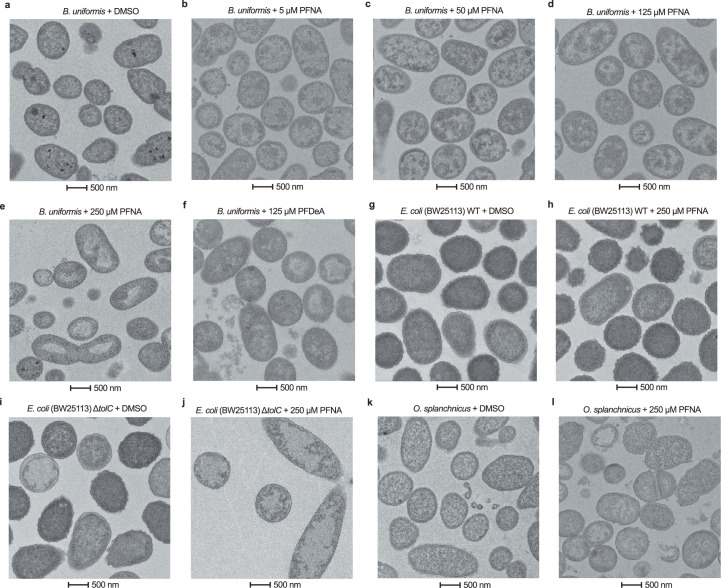
Transmission electron microscopy displays morphological changes upon PFAS exposure. **a-f**. TEM of *B*. *uniformis* cells grown in mGAM + DMSO (59 images from 3 biological replicate) **(a)**, 5 μM PFNA (31 images from 1 biological replicate) (**b**), 50 μM PFNA (40 images from 1 biological replicate) (**c**), 125 μM PFNA (51 images from 1 biological replicate) (**d**), 250 μM PFNA (79 images from 3 biological replicate) (**e**), or 125 μM PFDA (46 images from 1 biological replicate) (**f**) for 24 h. **g,h**. TEM of *E*. *coli*. BW25113 wild-type cells grown in mGAM + DMSO (40 images from 1 biological replicate) **(g)** or 250 μM PFNA (45 images from 1 biological replicate) **(h)** for 24 h. **i,j**. TEM of *E*. *coli*. BW25113 ∆*tolC* cells grown in mGAM + DMSO (31 images from 1 biological replicate) **(i)** or 250 μM PFNA (50 images from 1 biological replicate) **(j)** for 24 h. **k,l**. TEM of *O*. *splanchnicus* cells grown in mGAM + DMSO (37 images from 1 biological replicate) **(k)** or 250 μM PFNA (38 images from 1 biological replicate) **(l)** for 24 h. A tomogram for *B*. *uniformis* cells exposed to 250 µM PFNA confirming that the structures are inside the cells is available as a supplementary video.

**Extended Data Fig. 5:**
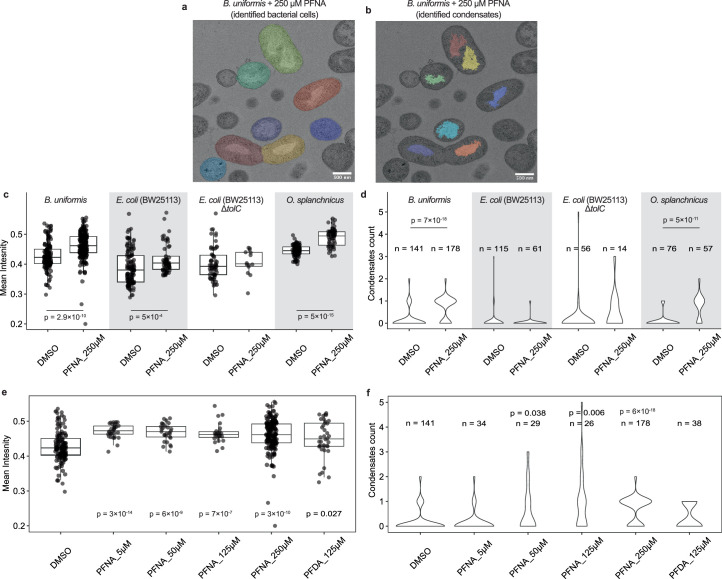
Morphological data supports intra-cellular accumulation of PFAS. **a-f**. Bacteria show distinct morphological features in transmission electron microscopy (TEM). **a,b**. Automatic identification of bacterial cells (**a**) and condensates (**b**) for *B*. *uniformis* cells grown in mGAM + 250 µM (116 mg/l) PFNA. 119 images analysed in total including all strains and exposures. **c**. Mean pixel intensity of bacterial cells show significant differences between DMSO and PFNA treated cells for *B*. *uniformis*, *E*. *coli* BW25113 and *O*. *splanchnicus*; two-sided t-test; p-values FDR corrected; Boxplot: centre = 50th percentile, bounds of box = 25th and 75th percentile, lower/upper whisker = lower/upper hinge −/+ 1.5 * inter quartile range; Number of cells analysed: *B*. *uniformis*: DMSO = 141, PFNA_250µM = 178; *E*. *coli* (BW25113): DMSO = 115, PFNA_250µM = 61; *E*. *coli* (BW25113) *∆tolC*: DMSO = 56, PFNA_250µM = 14; *O*. *splanchnicus*: DMSO = 76, PFNA_250µM = 57 (Supplementary Table 37). **d**. Condensate count per bacterial cell show significant increase in condensates for *B*. *uniformis* and *O*. *splanchnicus*, supporting morphological changes through PFNA exposure; two-sided t-test; p-values FDR corrected; Number of cells analysed: *B. uniformis*: DMSO = 141, PFNA_250µM = 178; *E*. *coli* (BW25113): DMSO = 115, PFNA_250µM = 61; *E*. *coli* (BW25113) *∆tolC*: DMSO = 56, PFNA_250µM = 14; *O*. *splanchnicus*: DMSO = 76, PFNA_250µM = 57 (Supplementary Table 37). **e**. *B*. *uniformis* data from **c** including data for 5, 50, 125 µM PFNA and 125 µM PFDA. Mean intensity of bacterial cells show significant differences between DMSO and PFNA or PFDA treated cells; two-sided t-test; p-values FDR corrected; Boxplot: centre = 50th percentile, bounds of box = 25th and 75th percentile, lower/upper whisker = lower/upper hinge −/+ 1.5 * inter quartile range; Number of cells analysed: DMSO = 141, PFNA_5µM = 34, PFNA_50µM = 29, PFNA_125µM = 26, PFNA_250µM = 178, PFDA_125µM = 38 (Supplementary Table 37. **f**. *B*. *uniformis* data from **d** including data for 5, 50, 125 µM PFNA and 125 µM PFDA. Condensate count per bacterial cell show significant increase in condensates for *B*. *uniformis* exposed to 50, 125 and 250 µM PFNA, supporting morphological changes through PFNA exposure; two-sided t-test; p-values FDR corrected; Number of cells analysed: DMSO = 141, PFNA_5µM = 34, PFNA_50µM = 29, PFNA_125µM = 26, PFNA_250µM = 178, PFDA_125µM = 38 (Supplementary Table 37).

**Extended Data Fig. 6:**
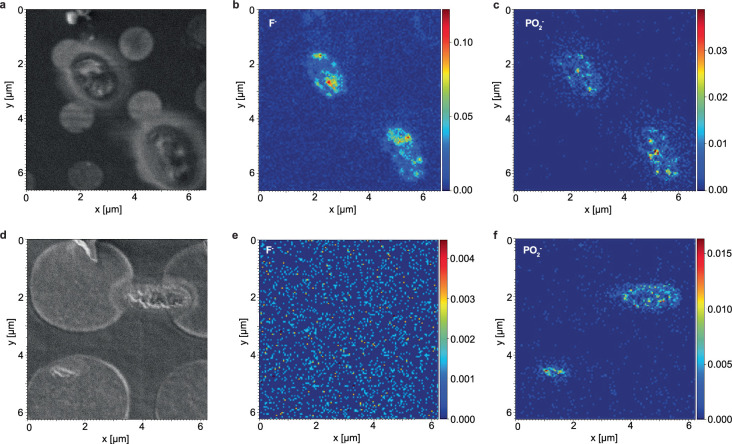
Cryogenic FIB-SIMS of E. coli ∆tolC exposed to 250 µM PFNA show intra-cellular accumulation of PFAS compared to the DMSO control. During cryogenic FIB-SIMS imaging, the vitrified sample is scanned by a Gallium focused ion beam (FIB) and time-of-flight secondary electron mass spectrometry (ToF-SIMS) is performed on the secondary ions created by the interaction of ion beam and sample at each scanning position. Thus, the chemical composition of the sample can be visualised pixel-by-pixel. **a-c**. *E*. *coli ∆tolC* exposed to 250 µM PFNA. **d-f**. *E*. *coli ∆tolC* exposed to DMSO control. For both cases, a spatial image of a cell resulting from the secondary electrons created during FIB imaging is shown on the left (**a,d**). Please note that because the specimens were frozen on a holey cryo-EM grid, round holes in the film are additionally observed, in addition to the elongated cells. On the right (**b,c,e,f**), ToF-SIMS images of the same cell are shown for mass-to-charge ratios 19 and 63, corresponding to F^−^ and PO_2_^−^, respectively. The colour bars indicate ion count, that is, regions shown in red are those where the most ions of the particular species were detected. F^−^ signal indicates the presence of PFNA, PO_2_^−^ was chosen as a comparison as it occurs in most bacterial cells and hence is a reliable marker for cells in negative SIMS imaging mode. In *E*. *coli ∆tolC* cells exposed to PFNA, a strong F^−^ signal is observed within the cell (**b**), which is not the case for the DMSO treated cells (**e**), in which a faint background signal can only be observed when adjusting the colour scale by several orders of magnitude.

Intracellular PFNA concentration in *B. uniformis* at 250 µM (116 mg l^−1^) exposure level would be circa 5–10 mM (2.3–4.6 g l^−1^), well above that of most native metabolites^46^. At these concentrations, the observed degree of bioaccumulation, if considered to be membrane associated only, would imply one molecule of PFNA per two lipid molecules in the cell membrane, which is physiologically improbable. How do cells cope with such high intracellular levels of highly effective surfactants such as PFAS and maintain their growth? To begin to answer this, we used transmission electron microscopy (TEM) to visualize *B.* *uniformis*, *O. splanchnicus*, *E. coli* wild type and *E. coli* ∆*tolC* mutant exposed to PFNA. Consistent with their observed levels of bioaccumulation, all cells except *E. coli* wild type showed disruption and condensation of cytoplasmic content (Extended Data Fig. 4). Similar morphological features have previously been observed in *Clostridium* *perfringens* exposed to lauric acid, which also has surfactant properties^47^. We quantified cytoplasmic condensation using automated image processing (Methods). *B.* *uniformis* and *O.* *splanchnicus* showed significantly increased mean pixel intensity and number of condensates upon PFNA and PFDA exposure (Extended Data Fig. 5). However, TEM imaging can show artefacts due to extensive sample processing, including resin embedding. Therefore, to confirm PFNA localization inside the bacterial cells, we performed cryogenic focused ion beam time-of-flight secondary ion mass spectrometry (FIB-SIMS) imaging of *E. coli ∆tolC* cells exposed to 250 µM PFNA. This method circumvents the need to embed the specimen in resin, preserving the cells in a near-native state in vitreous ice. Voxel-by-voxel three-dimensional analysis of the bacterial cell composition uncovered a strong fluorine signal localized inside the bacterial cells, corresponding to intracellular PFNA bioaccumulation (Fig. 3 and Extended Data Fig. 6). In total, 2 biological replicates were analysed, comprising 120 *E. coli ∆tolC* cells exposed to 250 µM PFNA and 44 control cells. All 120 cells exposed to PFNA showed a clear fluorine signal within the bacterial cell, while 43 control cells showed no fluorine signal, and one control cell showed a very low fluorine signal. Furthermore, intracellular PFNA was unevenly distributed with a distinct tendency for aggregation. The intracellular aggregation suggests either interaction of PFAS with cytosolic contents, such as proteins and other macromolecules, or phase separation of PFNA and cytosolic contents within the bacterial cell.

### Bacterial adaptation to PFAS in experimental evolution

**Extended Data Fig. 7:**
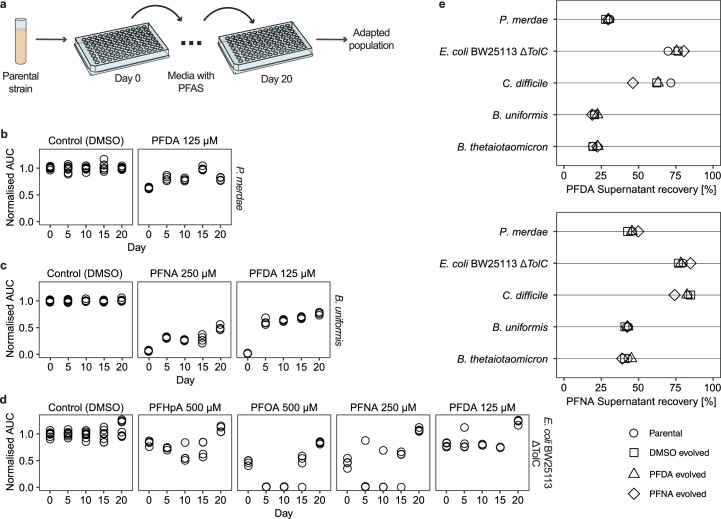
PFAS tolerance and bioaccumulation following adaptive laboratory evolution. **a**. Five gut bacterial species were evolved through serial passaging in growth medium containing one of four PFAS compounds (500 μM PFHpA, 500 μM PFOA, 250 μM PFNA, 125 μM PFDA) over 20 days. **b**. Improved growth of adapted *P*. *merdae* in presence of 125 μM PFDA at days 5, 10, 15 and 20 compared to day 0 (day 20: 1.3-fold change, p-value = 0.001). n = 4 independent populations per compound (Supplementary Tables 38,39). **c**. Improved growth of adapted *B*. *uniformis* population in presence of 250 μM PFNA and 125 μM PFDA after 5, 10, 15 and 20 days compared to day 0 (day20 PFNA 7-fold change, p-value = 0.0004; day 20 PFDA 46-fold change, p-value = 0.00003). n = 4 independent populations per compound (Supplementary Tables 38,39). **d**. Improved growth of adapted *E*. *coli* BW25113 *ΔTolC* population in presence of 500 µM PFHpA, 500 µM PFOA, 250 μM PFNA and 125 μM PFDA at day 20 compared to day 0 (PFHpA 1.3-fold change, p-value = 0.002; PFOA 1.7-fold change, p-value = 0.0006; PFNA 2.3-fold change, p-value = 0.0006; PFDA 1.6-fold change, p-value = 0.00005). n = 4 independent populations per compound (Supplementary Tables 38,39). **e**. Adapted populations retain PFDA and PFNA bioaccumulation capability. Mean of n = 4 independent populations per compound (Supplementary Table 40).

We next reasoned that if the cells had mechanisms to modulate bioaccumulation and/or to cope with high intracellular levels, resistance to inhibitory PFAS exposure would rapidly evolve under natural selection. We therefore evolved, through serial transfer, *B. uniformis*, *B.* *thetaiotamicron*, *P. merdae*, *C. difficile* and *E. coli ΔtolC* in a medium with 500 μM (182 mg l^−1^) PFHpA, 500 μM (207 mg l^−1^) PFOA, 250 μM (116 mg l^−1^) PFNA or 125 μM (64 mg l^−1^) PFDA (Extended Data Fig. 7a). Within 20 transfers (20 days)—corresponding to circa 100 generations—*P.* *merdae* (PFDA), *B. uniformis* (PFNA, PFDA) and *E. coli ΔtolC* (all tested PFAS) showed between 1.3- and 46-fold increase in growth (Extended Data Fig. 7b–d). Notably, the evolved cells retained the bioaccumulation capability of the respective parental strains (Extended Data Fig. 7e). Genome sequencing of evolved *B.* *uniformis* populations identified 55 variants linked to PFNA and PFOA exposure (Supplementary Table 4). More than half of the variants are in non-coding regions, suggesting that changes in gene expression contribute to the adaptation of *B.* *uniformis* to PFAS.

### Proteomic and metabolic changes following bioaccumulation

**Fig. 4:**
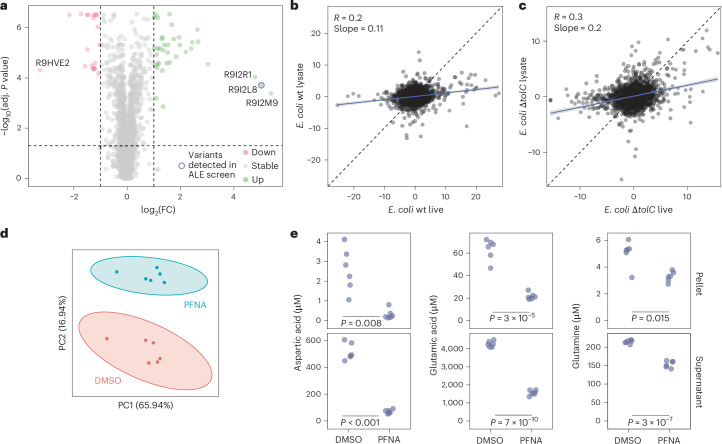
Bioaccumulation of PFNA affects bacterial physiology. **a**, Proteomics analysis shows proteins that are differentially abundant between *B.* *uniformis* treated with 20 µM PFNA and those treated with DMSO. The red and green dots mark proteins with a log_2_(abundance ratio) > 1 or < −1 (that is, twofold increase or decrease) and a multiple-testing corrected *P* value of less than 0.05; *n* = 6 biological replicates (Supplementary Table 24). The circle marks a protein from the RND efflux system (gene ID 962 corresponds to protein R9I2L8), for which nine missense variants within the coding region of the gene were identified in populations evolved under high PFAS concentrations (Supplementary Table 4). *P* values were calculated using analysis of variance followed by Benjamini–Hochberg correction for multiple testing. **b**,**c**, TPP analysis of *E. coli* BW25113 wt (wild type; low-PFNA bioaccumulating) (**b**) and *E. coli* BW25113 *ΔtolC* (high-PFNA bioaccumulating) (**c**). Lysate and live cells incubated with PFNA look more similar for *E. coli* BW25113 *ΔtolC* mutant compared with the wild type, supporting increased bioaccumulation in *ΔtolC* mutants. Each data point represents the summed log_2_(FC) across all temperatures for a specific protein. Black dashed line, diagonal; blue line, linear regression with 95% confidence interval (Supplementary Table 25). **d**, Principal component (PC) analysis shows a clear distinction between *B.* *uniformis* pellet samples treated with 20 µM PFNA and the control; *n* = 6 biological replicates (Supplementary Table 26). **e**, Aspartic acid, glutamic acid and glutamine concentrations in *B.* *uniformis* pellet and supernatant samples; *n* = 6 biological replicates (Supplementary Table 26). *P* values were calculated using two-sided *t*-test and corrected for multiple testing using the Benjamini–Hochberg method.

**Extended Data Fig. 8:**
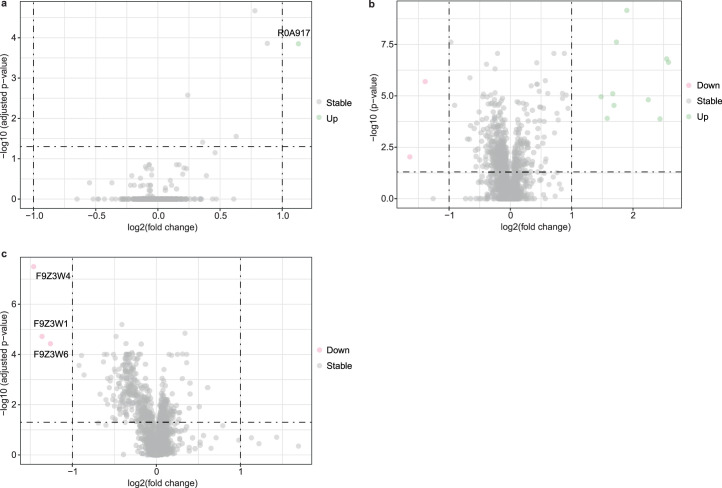
Proteomics results show effects of PFNA on abundance of proteins. **a-c**. Results showing the proteins that are differentially abundant between (**a**) *E*. *coli* BW25113, (**b**) *E*. *coli* BW25113 *ΔTolC*, and (**c**) *O*. *splanchnicus* treated with 20 µM PFNA in comparison to DMSO. The green and the blue dots mark proteins with a log2 abundance ratio >1 or <−1 (that is, two-fold increase or decrease) and a multiple-testing corrected p-value of less than 0.05. n = 6 biological replicates (Supplementary Table 24).

To characterize how intracellular localization of PFNA affects the molecular physiology of the bioaccumulating cells, we measured their proteomic and metabolic response. The proteome profile of the bioaccumulating *E.* *coli ΔtolC* mutant exposed to PFNA showed more changes than the non-accumulating wild type. Most PFNA-impacted proteins are either membrane- or stress response-related (Extended Data Fig. 8). *B. uniformis* cells exposed to PFNA also showed changes in membrane-related proteins, with the top three being efflux pumps, viz. R9I2M9 efflux transporter resistance–nodulation–cell division (RND) family, R9I2R1 NodT family efflux transporter and R9I2L8 hydrophobe/amphiphile efflux-1 (HAE1) family RND transporter (Fig. 4a). Missense variants of the latter RND transporter (R9I2L8) were also identified in all four PFNA-evolved *B.* *uniformis* populations with high (>0.92) allele frequencies. The proteomic and genomic changes together show the involvement of efflux pumps in *B.* *uniformis* in cellular interaction with PFAS, as we also found for *E. coli*. To identify proteins interacting with PFNA, we used thermal proteome profiling (TPP), which allows proteome-wide assessment of structural changes (stabilization or destablization) upon ligand binding^48,49^. We thus compared *E. coli* wild type and *ΔtolC* mutant exposed to PFNA, with the exposure before or after cell lysis, with the latter removing the membrane barrier for PFNA–protein interactions. The bioaccumulating *ΔtolC* mutant featured 10-fold more PFNA-interacting proteins (Supplementary Table 5). Furthermore, the thermal proteomic responses in the lysate and the live cells are more similar for the mutant than for the wild type, consistent with increased intracellular PFNA bioaccumulation in the *ΔtolC* mutant (Fig. 4b,c).

**Extended Data Fig. 9:**
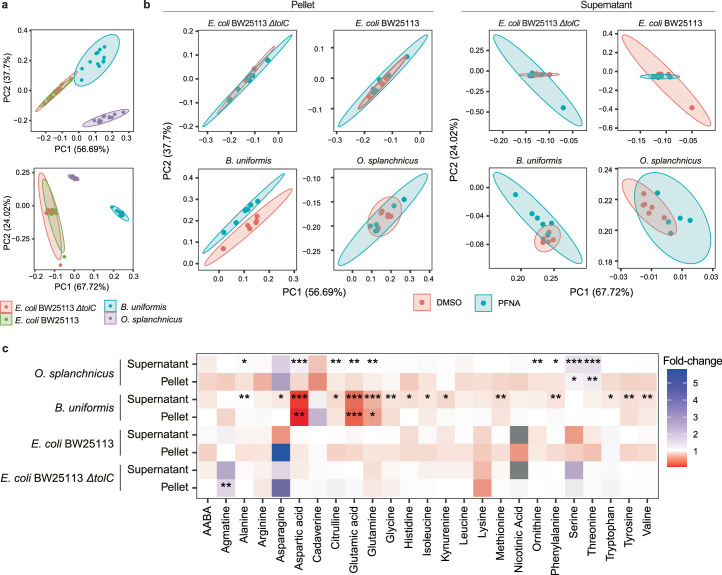
Effects of PFNA on abundance of metabolites in pellet and supernatant samples. **a**. PCA analysis shows clear distinction between bacterial strains, with *E*. *coli* wild-type and TolC mutant overlapping. n = 6 biological replicates (Supplementary Table 26). **b**. PCA analysis of pellet and supernatant samples shows clear distinction of PFNA and DMSO treated cells only for *B*. *uniformis* pellet samples (*B*. *uniformis* pellet PCA is a replicate of Fig.4d, added for completion). n = 6 biological replicates (Supplementary Table 26). **c**. Metabolomics analysis show change in intra- and extracellular metabolites. Fold change of analysed metabolites in supernatant and pellet samples of 20 µM PFNA exposed cells compared to control (DMSO) samples. Significance: * p<0.05, p<0.01, * p<0.001. P-values calculated using two-sided t-test and Benjamini-Hochberg correction for multiple testing. n = 6 biological replicates (Data and p-values provided in Supplementary Table 41).

The proteomic changes in efflux pump and other membrane-related proteins could be expected to result in altered levels of cellular metabolites. To test this, we used a targeted metabolomics method aimed at broadly conserved metabolites, including amino acids and vitamins. Metabolic changes were observed only for the bioaccumulating strains. *B. uniformis*, one of the highest bioaccumulators, showed a distinct metabolic response to PFNA exposure (Fig. 4d,e and Extended Data Fig. 9). Metabolites with altered levels include amino acids aspartate, glutamate and glutamine, and kynurenine, a metabolite implicated in the gut–brain axis^50,51^. Increased cadaverine levels, along with decreased glutamate and glutamine levels, indicate *B.* *uniformis* response like that observed in acid stress^52–54^.

### Gut bacteria impact PFNA faecal excretion in mice

**Fig. 5:**
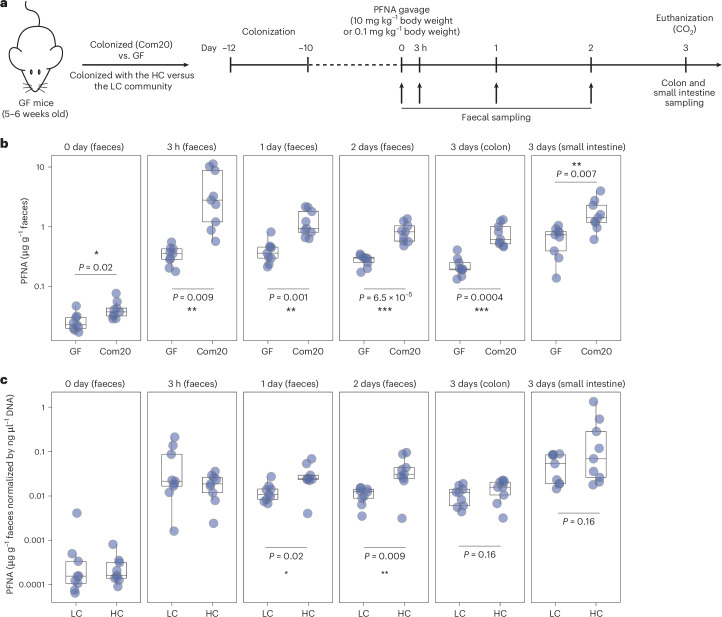
PFNA levels in the mouse faeces and gastrointestinal tract are microbiota dependent. **a**, Experimental setup for comparison of faecal excretion between GF mice and mice colonized with human gut bacteria (Com20) or comparison between mice colonized with high-PFNA bioaccumulating gut bacteria (HC) and mice colonized with low-PFNA bioaccumulating gut bacteria (LC). **b**, Mice colonized with a community of 20 human gut bacterial strains (Com20) show higher PFNA excretion after 10 mg kg^−1^ body weight PFNA exposure compared with GF controls. Box plot: centre = 50th percentile, bounds of box = 25th and 75th percentiles, lower and upper whiskers = lower and upper hinges ± 1.5 × interquartile range; two-sided *t*-test; *P* values are FDR corrected; *n* = 9 mice per group (Supplementary Table 27). **c**, Mice colonized with a community of HC show higher PFNA excretion after 10 mg kg^−1^ body weight PFNA exposure compared with mice colonized with LC. Box plot: centre = 50th percentile, bounds of box = 25th and 75th percentiles, lower and upper whiskers = lower and upper hinges ± 1.5 × interquartile range; two-sided *t*-test; *P* values are FDR corrected; *n* = 9 mice per group (Supplementary Table 28). All *y*-axes are on log_10_ scale. In **b**,**c**, *P value < 0.05; **P value < 0.01; ***P value < 0.001.

**Extended Data Fig. 10:**
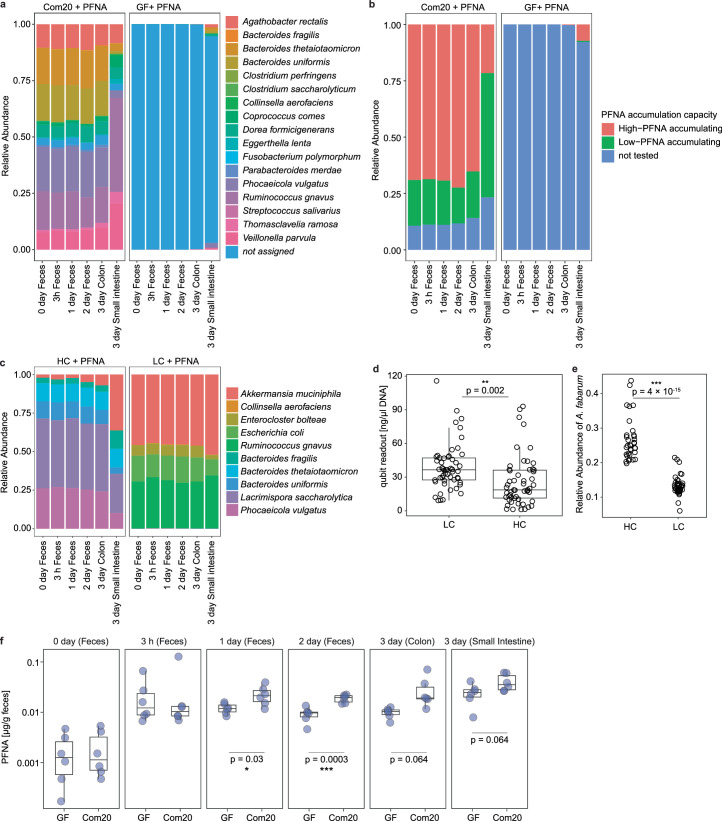
PFNA does not affect gut microbiota composition of mice colonised with human gut bacteria. **a**. Gut microbiota composition of mice colonised with 20 human gut bacterial strains (Com20) and exposed to 10 mg/kg body weight by oral gavage on day 0. 17 out of 20 gut bacteria colonised the mice with 7 strains making up 90% of bacterial composition (*P*. *vulgatus, R*. *gnavus, B*. *thetaiotaomicron, B*. *uniformis, A*. *rectalis, Veillonella parvula, Dorea formicigenerans*). The composition in the small intestine differed from that in the feces and colon, with the dominating species being *R*. *gnavus* and *V*. *parvula* (Supplementary Table 42). **b**. In the colon and fecal samples 70% of bacteria were classified as high-PFNA accumulating strains, while in the small intestine only 20% of bacteria were classified as high-PFNA accumulating (Supplementary Table 43). **c**. Gut microbiota composition of mice colonised with a community of five high-PFNA accumulating strains (HC) and five low-PFNA accumulating strains (LC) exposed to 10 mg/kg body weight by oral gavage on day 0. All gut bacteria colonised the mice, but *A*. *muciniphila* was present in both HC and LC colonised mice (Supplementary Table 44). **d**. LC (low-PFNA accumulating community) colonised mice showed higher DNA yields after DNA extraction compared to HC (high-PFNA accumulating community) colonised mice (1.96-fold change, p-value = 0.002), indicating higher bacterial content in LC colonised mice; two-sided t-test; Boxplot: centre = 50th percentile, bounds of box = 25th and 75th percentile, lower/upper whisker = lower/upper hinge −/+ 1.5 * inter quartile range; n = 54 samples per group (Supplementary Table 28). **e**. HC colonised mice showed higher relative abundance of *Acetobacter fabarum* spike-in compared to LC colonised mice (2-fold change, p-value = 0.0009), indicating higher bacterial content in LC colonised mice. Two-sided t-test; Boxplot: centre = 50th percentile, bounds of box = 25th and 75th percentile, lower/upper whisker = lower/upper hinge −/+ 1.5 * inter quartile range; Subset of 76 samples (Supplementary Table 45). **f**. Mice colonised with a community of 20 human gut bacterial strains (Com20) show higher PFNA excretion after 0.1 mg/kg body weight PFNA exposure compared to germfree (GF) controls; Boxplot: centre = 50th percentile, bounds of box = 25th and 75th percentile, lower/upper whisker = lower/upper hinge −/+ 1.5 * inter quartile range; two-sided t-test; p-values are FDR corrected; n = 6 mice per group; y-axes are on log_10_ scale (Supplementary Table 46).

To determine whether bacterial PFAS bioaccumulation occurs in an in vivo context, we tested C57BL/6J mice colonized with a community of 20 human gut bacterial strains (Com20)^55^ (Supplementary Table 6) against germ-free (GF) controls. The animals were administered a one-time oral dose of PFNA (10 mg kg^−1^ body weight) by gavage. This initial dose was selected based on previous reports indicating it causes no adverse effects in mice^56^, while still allowing for reliable detection in their faeces. Faecal samples were collected over the following 2 days, and on day 3, colon and small intestine content samples were taken post-mortem (Fig. 5a). Faecal microbiota analysis using 16S rRNA sequencing showed that 17 of the 20 strains colonized the mice and PFNA treatment had no effect on the microbiota composition (Extended Data Fig. 10a,b). Colonized mice showed a significantly higher faecal PFNA concentration at all follow-up time points (3 h, *P* = 0.009, fold change (FC) = 7.9; 1 day, *P* = 0.001, FC = 2.6; 2 days, *P* < 0.0001, FC = 2.8; 3 days (colon), *P* = 0.0004, FC = 3.1; 3 days (small intestine), *P* = 0.007, FC = 1.9) (Fig. 5b). Next, we tested whether microbial communities containing high- versus low-accumulating species would cause differences in faecal PFNA excretion. GF mice were colonized with a community of either five high- or five low-PFNA-accumulating strains (LC: *Akkermansia muciniphila*, *Collinsella aerofaciens*, *Enterocloster bolteae*, *E.* *coli* and *Ruminococcus gnavus*; HC: *Bacteroides fragilis*, *B. thetaiotaomicron*, *B.* *uniformis*, *Lacrimispora saccharolytica* and *Phocaeicola vulgatus*; Supplementary Table 6). PFNA treatment had no effect on gut microbiota composition (Extended Data Fig. 10c); however, LC-colonized mice showed circa twofold higher colonization compared with HC-colonized mice (Extended Data Fig. 10d,e). HC-colonized mice showed increased faecal excretion of PFNA, normalized by bacterial biomass, on days 1 and 2 (1 day, *P* = 0.02, FC = 2.4; 2 days, *P* = 0.009, FC = 3.8) (Fig. 5c). We also carried out a GF versus colonized comparison using a 100× lower PFNA dose (0.1 mg kg^−1^ body weight PFNA), which showed the same trend of increased excretion for the colonized mice (Extended Data Fig. 10f). Together, the mouse experiments show the contribution of gut bacterial bioaccumulation to faecal PFAS excretion.

## Discussion

Our study reveals the capacity of gut bacteria to bioaccumulate PFAS and has fundamental implications for understanding how PFAS interact with biological systems. We find that bioaccumulation is characterized by high capacity and rapid kinetics. We observe >50-fold enrichment of PFAS within bacterial pellets implying intracellular concentration in the mM range. The internalization of PFAS is supported both by analytical and biological evidence. The latter includes multi-omics analyses, imaging, phylogenetic grouping of bioaccumulators and increased bioaccumulation in the *E. coli* efflux-pump mutant.

How do the bioaccumulating cells maintain their viability and growth given the surfactant-like properties of PFAS? Cryogenic FIB-SIMS imaging shows that the bioaccumulated molecules aggregate in dense clusters (Fig. 3 and Supplementary Fig. 2). Sequestration of PFAS into dense intracellular aggregates appears to minimize interference with vital cellular processes, which may explain the bacterial viability and growth. This is supported by the relatively limited impact of PFAS on the bacterial proteome and metabolome. Yet, these changes may have implications in a community or host context. We observe changes in the expression of membrane proteins, including transporters, and reduced the secretion of amino acids that can reduce cross-feeding opportunities in microbial communities^57^. Studies in synthetic and ex vivo communities would be needed to investigate how PFAS-induced changes in bacterial physiology impact fellow community members. Redistribution of bioaccumulated PFAS following cell division also remains an open question. FIB-SIMS analysis of growing cells in the presence of PFAS could help elucidate the redistribution of PFAS during cell division.

It is unlikely that bacteria have evolved a specific tolerance mechanism for PFAS given the relatively low fitness cost and complex selection pressures in the gut environment. We postulate that the cellular entry and cytosolic aggregation are consequences of the physicochemical properties of PFAS allowing interaction with non-membrane cellular components. The interaction of PFAS with proteins has been noted in the case of human serum^58^. Furthermore, studies in aqueous solution have shown self-aggregation of PFAS molecules^59^. Our conjecture is further supported by the observation that bioaccumulation capacity is phylogenetically segregated and could be genetically modulated in *E. coli*. Another indication for the key role of PFAS biophysical properties is the positive correlation between chain length and the bioaccumulation (Fig. 1g). In support, a recent study investigating the kinetics of PFAS compounds ingested by a volunteer found that long-chain PFAS bioaccumulate most in the human body^37^. Together, the biophysical considerations and our data suggest that interactions with transporters and self-aggregation in the cytosol, possibly facilitated by binding to proteins, underlie the observed PFAS condensates inside the bacterial cells. Thus, PFAS bioaccumulation inside the cells is likely to be a consequence of biophysical properties of PFAS rather than an active, regulated, bacterial process.

A key open question is the mechanism of PFAS uptake. Given their surfactant-like properties as well as our data showing active export in *E. coli*, passive diffusion across the membrane appears unlikely. There is no correlation between PFAS bioaccumulation by gut bacteria and the previously observed drug bioaccumulation data^36^, indicating distinct import mechanisms. Indeed, higher PFAS bioaccumulation by Gram-negative bacteria is in contrast with their generally lower susceptibility to drug and xenobiotic uptake^60,61^. The rapid kinetics of PFAS uptake by gut bacteria (timescale of minutes; Extended Data Fig. 2f) contrasts with the slower accumulation reported in environmental bacteria, which occurs over days^29^. This difference may reflect differences in bacterial membrane composition, surface properties, or transporter properties and expression. Thus, identifying gut bacterial transporters involved in PFAS import through, for example, functional genomic approaches, will be critical to fully elucidate microbial PFAS sequestration.

By uncovering microbial PFAS bioaccumulation in molecular detail, this study provides a framework for investigating microbial contribution to PFAS toxicokinetics. Our gnotobiotic mouse studies show that gut bacteria bioaccumulate PFAS in an in vivo context. However, these experiments were done using a one-time dosage while most populations experience low chronic exposure. Therefore, cohort studies tracking PFAS intake, blood levels, urine and faecal excretion, and microbiota composition over a prolonged period will be an important next step.

## Methods

### Bacterial strains and cultivation

Strains were selected to represent prevalent and abundant members of the healthy human gut microbiota^34,35^ (Supplementary Tables 1 and 3). *E. coli* mutants were obtained from the Typas laboratory (EMBL Heidelberg, BW25113 wild type, BW25113 ∆*tolC*, BW25113 *imp4213::FRT*) and Luisi laboratory (University of Cambridge, C43 (DE3) wild type, C43 (DE3) ∆*acrAB-tolC*, C43 (DE3) ∆*acrAB*). All bacterial experiments were performed in an anaerobic chamber (Coy Laboratory Products) filled with 2% hydrogen and 12% carbon dioxide in nitrogen. The chamber was equipped with a palladium catalyst system for oxygen removal, a dehumidifier and a hydrogen sulfide removal system. Bacteria were grown at 37 °C in modified Gifu anaerobic medium (mGAM, HyServe, produced by Nissui Pharmaceuticals), prepared according to the instructions from the manufacturer and sterilized by autoclaving. Bacteria for starting cultures were grown for 1 or 2 days (depending on the growth rate) in 10 ml of media in 15-ml plastic tubes, which were inoculated directly from frozen glycerol stocks. Cultures were then diluted 100-fold and incubated again for the same amount of time before the start of the experiments. Unless otherwise specified, the screening plates and tubes with cultivation medium were prepared the day before at 2× compound concentration (2% DMSO) and placed into the chamber overnight to ensure anaerobic conditions for inoculation. Inoculation was performed 1:1 with a bacterial culture, and plates were sealed with AlumaSeal II film (A2350-100EA) to avoid evaporation during incubation.

### Standard compounds

For all measured compounds, including PFHpA, PFOA, PFNA and PFDA, pure standards were obtained from Sigma-Aldrich (Merck KGaA). All compounds were dissolved in DMSO, with the exception of NMOR, MDMA and methamphetamine, which came dissolved in methanol, and cocaine and heroin, which came dissolved in acetonitrile. A mix containing 14 PFAS compounds was purchased from Agilent (ITA-70). Labelled standards of PFNA and a mix of 13 PFAS standards were purchased from Cambridge Isotope Laboratories and Greyhound Chromatography (CLM-8060-1.2, MPFAC-C-ES).

### Community-based screening approach

On the day of the screen, communities were assembled by pooling together second passages of individual strains according to their OD_600_ values (Supplementary Table 1). Assembled communities were then centrifuged at 25 °C and 3,202 × *g* for 15 min, and the pellet was resuspended in PBS buffer to create a community with OD_600_ of 7.5 (Extended Data Fig. 1a,b). Each well was inoculated 1:1 with the community in PBS to reach a starting OD_600_ of 3.75 and a compound concentration of 20 µM (1% DMSO). For compound control wells, bacteria-free PBS was added to the respective wells. Plates were incubated at 37 °C for 4 h, after which they were centrifuged at 21 °C and 3,202 × *g* for 15 min. The supernatant and compound controls were transferred to fresh 96-well plates and stored at −80 °C until extraction.

### Single strain-xenobiotic screen

Ten compounds selected based on the ‘Community-based screening approach’ were tested for sequestration by individual strains (Supplementary Table 7). On the day of the screen, each well was inoculated 1:1 with a second-passage culture to reach a starting OD_600_ of 0.05 and a compound concentration of 20 µM (1% DMSO) (Fig. 1a and Extended Data Fig. 1c). For compound control wells, bacteria-free mGAM was added to the respective wells. Plates were incubated at 37 °C for 24 h, after which they were removed from the anaerobic chamber for sample collection. Whole culture, supernatant and compound control samples were collected and stored at −80 °C until extraction.

### PFAS bioaccumulation analysis

#### Resting cell assay

Each well was inoculated 1:1 with a culture in PBS to reach the desired starting OD_600_ of 3.75, unless otherwise specified (Figs. 1b,c,g and 2c, and Extended Data Figs. 2a–d,g,i and 3a). For compound control wells, bacteria-free PBS was added to the respective wells. Samples were incubated at 37 °C for 4 h, after which they were removed from the anaerobic chamber for sample collection. Whole culture, supernatant and compound control samples were collected and stored at −80 °C until extraction.

#### Growth assay

On the day of the screen, each well was inoculated 1:1 with a second-passage culture to reach a starting OD_600_ of 0.05 (Fig. 1f and Extended Data Fig. 2h). For compound control wells, bacteria-free mGAM was added to the respective wells. Samples were incubated at 37 °C for 24 h, after which they were removed from the anaerobic chamber for sample collection. Whole culture, supernatant and compound control samples were collected and stored at −80 °C until extraction.

#### PFAS time-course experiment with growing *B. uniformis* culture

On the day of the screen, each tube was inoculated 1:1 with a second-passage culture to reach a starting OD_600_ of 0.05 and a concentration of 20 µM (9.3 mg l^−1^) PFNA (Fig. 1d and Extended Data Fig. 2e). Samples were incubated at 37 °C for 11 h. Bacteria-free mGAM was added to compound control samples. OD_600_ was measured every hour, and supernatant, whole culture and compound control samples for PFNA analysis were collected also every hour and stored at −80 °C until extraction.

#### PFAS time-course experiment with resting *B. uniformis* culture

On the day of the screen, each well was inoculated 1:1 with a culture in PBS to reach a starting OD_600_ of 3.75 and a concentration of 20 µM (9.3 mg l^−1^) PFNA (Fig. 1e). Samples were incubated at 37 °C. Bacteria-free PBS was added to compound control samples. Supernatant, pellet, whole culture and compound control samples for PFNA analysis were collected at 0 h, 4 h, 1 day, 2 days, 4 days and 7 days and stored at −80 °C until extraction. The aim of this experiment was to see whether *B. uniformis* would release PFAS after a prolonged period irrespective of the viability or cell death. Therefore, the viability of the *B. uniformis* culture was not assessed.

#### PFAS time-course experiment with stationary-phase *B. uniformis* culture

A total of 1.5 ml of stationary second-passage cultures of *B. unifomis* or pure mGAM was spiked with 15 μl of 2 mM PFNA in DMSO (final concentration 20 µM (9.3 mg l^−1^) PFNA, 1% DMSO) (Extended Data Fig. 2f). Whole culture, supernatant and compound control samples were collected at 0, 15, 30 and 60 min and stored at −80 °C until extraction.

#### PFAS accumulation in live, heat-inactivated and lysed bacterial cultures

On the day of the screen, each well was inoculated 1:1 with a live, heat-inactivated or lysed culture in PBS to reach a starting OD_600_ of 3.75 (Fig. 2a). Second-passage cultures were spun down, and the pellet was resuspended in PBS to an OD_600_ of 7.5. Each culture was split up into three aliquots: live, heat inactivated and lysed cultures. Live cultures were used as is. Bacteria were heat inactivated at 70 °C for 40 min, and lysed cultures were additionally freeze-thawed three times and sonicated for 3 min. After the respective cultures or bacteria-free PBS was added to the respective wells, the plates were sealed and incubated at 37 °C. After 4 h, whole culture, supernatant and compound control samples were collected and stored at −80 °C until extraction.

#### Low-concentration experiment (160 ng l^−1^)

Three biological replicates of *B. uniformis* were grown over the course of 2 days (Fig. 2d and Extended Data Fig. 3c,d). On the day of the experiment, the OD_600_ of the second-passage cultures was measured, and the equivalent of 2 × 0.5 l of OD_600_ of 3.75 of each replicate was centrifuged at 3,202 × *g* for 15 min and the supernatant removed. One pellet per biological replicate was kept as negative control and stored at −80 °C until extraction. The other pellet was resuspended in 0.5 l of 160 ng l^−1^ PFNA in PBS and incubated at 37 °C for 1 h in HDPE bottles (Buerkle 10531712). A total of 3 × 0.5 l of 160 ng l^−1^ PFNA in PBS without a bacterial pellet was used as the compound control and incubated for the same amount of time. For collection, samples were removed from the anaerobic chamber and centrifuged at 3,202 × *g* for 15 min. Supernatant and pellet samples were stored separately at −80 °C until extraction.

#### PFHpA, PFOA, PFNA and PFDA pellet recovery

To determine pellet concentration based on pellet weight across four PFAS compounds (PFHpA, PFOA, PFNA, PFDA), a resting cell assay and growth assay were conducted at an exposure concentration of 5 µM in 0.5 ml volume (Fig. 2e and Extended Data Fig. 3e). On the day of the screen, each well was inoculated 1:1 with a second-passage culture to reach a starting OD_600_ of 0.05 in mGAM or a starting OD_600_ of 3.75 in PBS and a starting concentration of 5 µM of the respective PFAS compound (0.5 ml total volume). Samples were incubated at 37 °C for 24 h (mGAM) or 37 °C for 4 h (PBS), after which they were removed from the anaerobic chamber for sample collection. Supernatant was collected for LC–MS analysis, and the pellets were weighed and also collected for LC–MS analysis.

In the resting assay, *B.* *uniformis* and *E.* *coli* pellets weighed ~8 mg and ~5 mg, respectively, meaning bacterial cells contribute only circa 1–2% to total culture weight and volume.

#### Bioaccumulation of a mix of 14 PFAS compounds

On the day of the screen, each well was inoculated 1:1 with a culture in PBS to reach a starting OD_600_ of 3.75 (1 mg l^−1^ for each PFAS compound: perfluorobutanoic acid (PFBA), perfluorobutanesulfonic acid (PFBS), perfluoropentanoic acid (PFPeA), perfluorohexanoic acid (PFHxA), PFHxS, PFHpA, PFOA, PFOS, PFNA, PFDA, PFUnDA, perfluorododecanoic acid (PFDoDA), perfluorotridecanoic acid (PFTrDA) and perfluorotetradecanoic acid (PFTA)) (Fig. 2f and Extended Data Fig. 3f). Bacteria-free PBS was added to the respective compound control wells. Samples were incubated at 37 °C for 4 h, after which they were removed from the anaerobic chamber for sample collection. Whole culture, supernatant, pellet and compound control samples were collected and stored at −80 °C until extraction.

#### PFNA and PFDA bioaccumulation by evolved populations

A total of 20 µl of a second-passage culture was added to 380 µl of PFAS in mGAM to reach a starting concentration of 20 µM PFNA or PFDA (Extended Data Fig. 7e). Bacteria-free mGAM was added to compound control samples. Samples were incubated at 37 °C for 24 h, after which whole culture, compound control and supernatant samples were collected for LC–MS analysis.

### PFAS solubility

For all PFAS assays, either mGAM or PBS with 1% DMSO was used. To test whether the solubility of PFAS compounds could affect our assays, we measured the solubility of PFNA and PFDA in mGAM (1% DMSO), PBS (1% DMSO) and 80% methanol (1% DMSO). PFNA was soluble in all conditions up to 500 µM, while PFDA was soluble up to 500 µM in 80% methanol (1% DMSO) and mGAM (1% DMSO), and up to 100 µM in PBS (1% DMSO) (Supplementary Fig. 1a).

### Sample extraction for LC–MS/MS

#### Bacterial samples

For the ‘Community-based screening approach’, 70 μl of supernatant was extracted with 140 μl of ice-cold methanol:acetonitrile (1:1) containing the internal standard (IS; 20 µM caffeine, 60 µM ibuprofen) and incubated at 4 °C for 30 min. For the ‘Single strain-xenobiotic screen’, a 50-μl sample was extracted with 200 μl of ice-cold methanol:acetonitrile (1:1) containing the IS (100 µM caffeine, 60 µM ibuprofen) and incubated at 4 °C for 15 min. For other follow-up in vitro assays, a 50-μl sample was extracted with 200 μl of ice-cold methanol:acetonitrile (1:1) containing the IS (60 µM ibuprofen or 20 µM caffeine) and incubated at 4 °C for 15 min. For the PFAS-mix assay, all samples were diluted 1:1 with water and 10 µl of diluted sample was extracted with 90 µl of ice-cold methanol:acetonitrile (1:1) containing the IS (final IS concentration 20 µg l^−1^ for each IS) at 4 °C for 15 min. Sample plates were then centrifuged at 4 °C and 3,202 × *g* for 10 min. Supernatants were transferred to 96-well plates for LC–MS analysis. Samples for concentration calibration and bacteria-free compound controls were processed in the same way.

#### Sample extraction per EPA Draft Method 1633

Supernatant and pellet samples were extracted according to the EPA 3rd Draft Method 1633 for analysis of PFAS in aqueous, solid, biosolid and tissue samples^44^ and the corresponding Agilent application note^45^ (Fig. 2d and Extended Data Fig. 3c,d). In short, 500 ml of supernatant or compound control samples were weighed and spiked with a known concentration of an IS (1 ml of 50 µg l^−1^ 13C9-PFNA). Pellet samples underwent three freeze–thaw cycles to ensure bacterial lysis. Samples were weighed, resuspended in 20 ml of 0.3% methanolic ammonium hydroxide, spiked with a known concentration of an IS (1 ml of 50 µg l^−1^ 13C9-PFNA) and shaken for 30 min. Samples were then centrifuged at 1,569 × *g* for 10 min, and the supernatant was transferred to a fresh sample tube. The remaining pellet was resuspended in 15 ml of 0.3% methanolic ammonium hydroxide, shaken for 30 min and centrifuged again. The supernatant was added to the same collection tube, and the pellet underwent the same process again using 10 ml of 0.3% methanolic ammonium hydroxide. To the combined supernatants from each pellet, circa 10 mg carbon (Agilent 5982-4482) was added. Samples were handshaken for up to 5 min and then centrifuged for 10 min at 1,569 × *g*. The supernatants were collected in a fresh sample tube. Agilent solid-phase extraction (SPE) cartridges (Agilent 5610-2150) were loaded with silanized glass wool (Agilent 8500-1572) fitted with large volume adaptors (Agilent 12131012 and 12131001) and conditioned with 15 ml of 1% methanolic ammonium hydroxide followed by 5 ml of 0.3 M formic acid. Samples were then loaded into SPE cartridges and set to a low flow rate of circa 5 ml min^−1^, followed by a rinse with 10 ml reagent water and 5 ml of 1:1 0.1 M aqueous formic acid:methanol, before being dried under the vacuum. Sample bottles were rinsed and eluted with 5 ml of 1% methanolic ammonium hydroxide. Then, 25 µl of acetic acid was added to each sample and each sample was vortexed. To each supernatant and compound control eluate, 10 mg carbon (Agilent 5982-4482) was added, and samples were handshaken for up to 5 min and then centrifuged for 10 min at 1,569 × *g*. All samples were filtered through a nylon syringe filter (Agilent 9301-6476, 5190-5092) into a collection tube for LC–MS analysis.

#### Mouse faecal samples

Frozen faecal samples were weighed out into tubes with beads, and 250 μl extraction buffer (methanol + 0.05 KOH + 15 μM caffeine (Fig. 5b) or 0.01 mg l^−1^ M9PFNA (Fig. 5c and Extended Data Fig. 10f)) was added. Tubes were then homogenized at 1,500 rpm (SPEX SamplePrep LLC) for 10 min followed by centrifugation at 16,900 × *g* and 4 °C for 5 min. Then, 20 μl supernatant was added to 80 μl water + 0.1% formic acid, and the mixture was vortexed, incubated at 4 °C for 15 min and centrifuged at 16,900 × *g* and 4 °C for 5 min. The supernatant was transferred to LC–MS vials with inserts. Samples for concentration calibration were processed in the same way.

### LC–MS/MS (QTOF) xenobiotic measurements

#### QTOF parameters

LC–MS analysis was performed on an Agilent 1290 Infinity II LC system coupled with an Agilent 6546 LC/Q-TOF. The quadrupole time-of-flight (QTOF) MS scan was operated in positive or negative MS mode using four different collision energies (0 V, 10 V, 20 V, 40 V) (30–1,500 m/z), depending on the xenobiotic targeted for measurement (Supplementary Table 2). The source parameters were as follows: gas temperature, 200 °C; drying gas, 9 l min^−1^; nebulizer, 20 psi; sheath gas temperature, 400 °C, sheath gas flow, 12 l min^−1^; VCap, 3,000 V; nozzle voltage, 0 V; fragmentor, 110 V; skimmer, 45 V; and Oct RF Vpp, 750 V. The online mass calibration was performed using a reference solution (positive: 121.05 and 922.01 m/z; negative: 112.99 and 1033.99 m/z). The compounds were identified based on their retention time, accurate mass and fragmentation patterns. For all measured compounds, pure standards were used for method development, compound identification and calibration.

Five different LC methods were applied.

#### 15-min reverse-phase LC method used with QTOF in positive ionization mode

The separation was performed using a ZORBAX RRHD Eclipse Plus column (C18, 2.1 × 100 mm, 1.8 μm; Agilent 858700-902) with a ZOBRAX Eclipse Plus (C18, 2.1 × 5 mm, 1.8 μm; Agilent 821725-901) guard column at 40 °C (Community-Based Screening Approach and Extended Data Fig. 1b). The multisampler was kept at a temperature of 4 °C. The injection volume was 1 μl and the flow rate was 0.4 ml min^−1^. The mobile phases consisted of A: water + 0.1% formic acid + 5 mM ammonium formate, and B: methanol + 0.1% formic acid + 5 mM ammonium formate. The 15-min gradient started with 5% solvent B, which was increased to 30% by 1 min and then further increased to 100% by 7 min and held for 3 min, before returning to 5% solvent B for a 5-min re-equilibration.

#### 10-min reverse-phase dual-pump LC method used with QTOF in positive ionization mode

The separation was performed using two ZORBAX RRHD Eclipse Plus columns (C18, 2.1 × 100 mm, 1.8 μm; Agilent 858700-902) with the ZOBRAX Eclipse Plus (C18, 2.1 × 5 mm, 1.8 μm; Agilent 821725-901) guard columns at 40 °C (Fig. 1a). The multisampler was kept at a temperature of 4 °C. The injection volume was 1 μl, and the flow rate was 0.4 ml min^−1^. The mobile phases consisted of A: water + 0.1% formic acid + 5 mM ammonium formate and B: methanol + 0.1% formic acid + 5 mM ammonium formate. The 10-min gradient started with 5% solvent B, which was increased to 30% by 1 min and then further increased to 100% by 7 min and held for 1.7 min, before returning to 5% solvent B at 8.8 min, which was held until 10 min. The re-equilibration gradient started with 5% solvent B, which was then ramped up to 100% solvent B at 0.1 min and held until 4 min before returning to the starting condition of 5% solvent B at 4.1 min.

#### 13-min reverse-phase LC method used with QTOF in negative ionization mode

The separation was performed using a ZORBAX RRHD Eclipse Plus column (C18, 2.1 × 100 mm, 1.8 μm; Agilent 858700-902) with ZOBRAX Eclipse Plus (C18, 2.1 × 5 mm, 1.8 μm; Agilent 821725-901) guard columns at 40 °C (Community-Based Screening Approach and Extended Data Fig. 1b). The multisampler was kept at a temperature of 4 °C. The injection volume was 1 μl, and the flow rate was 0.4 ml min^−1^. The 13-min gradient started with 35% solvent B, which was increased to 100% by 9 min and held for 1 min, before returning to 35% solvent B for a 3-min re-equilibration. Mobile phases for BPA, BPB, BPF and catechol analysis consisted of A: water and B: methanol; mobile phases for BPAF, BPS, PFNA, PFOA, patulin and 2-nitrofluorene analysis consisted of A: water + 5 mM ammonium acetate + 0.03% acetic acid and B: methanol + 5 mM ammonium acetate + 0.03% acetic acid.

#### 10-min reverse-phase dual-pump LC method used with QTOF in negative ionization mode

The separation was performed using two ZORBAX RRHD Eclipse Plus columns (C18, 2.1 × 100 mm, 1.8 μm; Agilent 858700-902) with the ZOBRAX Eclipse Plus (C18, 2.1 × 5 mm, 1.8 μm; Agilent 821725-901) guard columns at 40 °C (Figs. 1a,b,e,g and 2a,c and Extended Data Figs. 2f,g,i and 3a). The multisampler was kept at a temperature of 4 °C. The injection volume was 1 μl and the flow rate was 0.4 ml min^−1^. The mobile phases consisted of A: water + 5 mM ammonium acetate + 0.03% acetic acid and B: methanol + 5 mM ammonium acetate + 0.03% acetic acid. The 10-min gradient started with 35% solvent B, which was increased to 100% by 7 min and held for 1.7 min, before returning to 35% solvent B at 8.8 min, which was held until 10 min. The re-equilibration gradient started with 35% solvent B, which was then ramped up to 95% solvent B at 0.1 min and held until 4 min before returning to the starting condition of 35% solvent B at 4.1 min.

#### 2-min reverse-phase LC method used with QTOF in negative ionization mode

The separation was performed using a ZORBAX RRHD Eclipse Plus column (C18, 3.0 × 50 mm, 1.8 μm; Agilent 959757-302) at 40 °C (Extended Data Fig. 7e). The multisampler was kept at a temperature of 4 °C. The injection volume was 1 μl and the flow rate was 0.8 ml min^−1^. The mobile phases consisted of A: water + 5 mM ammonium acetate + 0.03% acetic acid and B: methanol + 5 mM ammonium acetate + 0.03% acetic acid. The 2-min gradient started with 30% solvent B, which was increased to 100% by 0.5 min and held until 1 min, before returning to 30% solvent B at 1.1 min until 2 min.

### LC–MS/MS (QQQ) PFAS measurements

#### QQQ parameters

LC–MS/MS analysis was performed on an Agilent 1290 Infinity II LC system coupled with an Agilent 6470 triple quadrupole. The triple quadrupole (QQQ) was operated in dynamic multiple reaction monitoring (dMRM) mode. The source parameters were as follows: gas temperature, 300 °C; gas flow, 10 l min^−1^; nebulizer, 50 psi; sheath gas temperature, 300 °C; sheath gas flow, 11 l min^−1^; VCap, 3,500 V (positive) or 3,000 V (negative); and nozzle voltage, 2,000 V (positive) or 500 V (negative). The transitions for PFAS can be found in Supplementary Table 29. Pure standards were used for method development, compound identification and calibration.

#### 2-min reverse-phase LC method used with QQQ

The separation was performed using a ZORBAX RRHD Eclipse Plus (C18, 3 × 50 mm, 1.8 μm; Agilent 959757-302) or a Poroshell 120 EC-C18, 1.9 μm, 2.1 × 50 mm (Agilent 699675-902) at 40 °C (Figs. 1b–d,f and 2e, and Extended Data Figs. 2a–f,h and 3e). The multisampler was kept at a temperature of 4 °C. The injection volume was 1 μl and the flow rate was 0.8 ml min^−1^. The mobile phases consisted of either A: water + 5 mM ammonium acetate + 0.03% acetic acid and B: methanol + 5 mM ammonium acetate + 0.03% acetic acid, or A: water + 0.1% formic acid and B: acetonitrile + 0.1% formic acid. The 2-min gradient started with 30% solvent B, which was increased to 100% by 0.5 min and held until 1 min, before returning to 30% solvent B at 1.05 min and held until 2 min.

#### 10-min reverse-phase LC method used with QQQ for mouse faecal sample analysis

The separation was performed using a ZORBAX RRHD Eclipse Plus column (C18, 2.1 × 100 mm, 1.8 μm; Agilent 858700-902) with a ZOBRAX Eclipse Plus (C18, 2.1 × 5 mm, 1.8 μm; Agilent 821725-901) guard column at 40 °C (Fig. 5 and Extended Data Fig. 10f). The multisampler was kept at a temperature of 4 °C. The injection volume was 2–10 μl (Fig. 5b: 2 µl; Fig. 5c: 10 µl; Extended Data Fig. 10f: 5 µl) and the flow rate was 0.4 ml min^−1^. The mobile phases consisted of A: water + 0.1% formic acid and B: acetonitrile + 0.1% formic acid. The 10-min gradient started with 5% solvent B, which was increased to 90% by 5 min and further increased to 100% solvent B by 7 min, before returning to 5% solvent B at 7.1 min and held until 10 min (Supplementary Fig. 1b).

### LC–MS/MS PFAS measurements per EPA Draft Method 1633

Samples were analysed according to the EPA 3rd Draft Method 1633 (ref. ^44^) and the corresponding Agilent application note^45^ (Fig. 2d,f and Extended Data Fig. 3d,f). In short, separation was performed using a ZORBAX Eclipse Plus column (C18, 2.1 × 100 mm, 1.8 μm; Agilent 959758-902) with a ZOBRAX Eclipse Plus (C18, 2.1 × 5 mm, 1.8 μm; Agilent 821725-901) guard column at 40 °C. A PFC delay column was used (Agilent 5062-8100, 4.6 × 30 mm). The multisampler was kept at a temperature of 4 °C. The injection volume was 5 μl and the flow rate was 0.4 ml min^−1^. The mobile phases consisted of A: 2 mM ammonium acetate in 95% water + 5% acetonitrile and B: acetonitrile. The 10-min gradient started with 2% solvent B, which was increased to 95% by 10 min, before returning to 2% solvent B for a 2-min re-equilibration. LC–MS/MS analysis was performed on an Agilent 1290 Infinity II LC system coupled with an Agilent 6470 triple quadrupole. The QQQ was operated in dMRM mode. The source parameters were as follows: gas temperature, 230 °C; gas flow, 6 l min^−1^; nebulizer, 20 psi; sheath gas temperature, 355 °C; sheath gas flow, 10 l min^−1^; VCap, 3,500 V (positive) or 2,500 V (negative); and nozzle voltage, 2,000 V (positive) or 0 V (negative). Pure standards (Agilent ITA-70) and labelled standards (Wellington Laboratories MPFAC-C-ES) were used for method development, compound identification and calibration (Supplementary Fig. 1c).

### LC–MS/MS for the analysis of PFNA

Analysis was performed (Imperial College London; Extended Data Fig. 3c) using a Shimdazu Nexera X2 LC-system coupled to an LCMS-8060 triple quadrupole system with an electrospray ionization source operated in negative ionization mode (Shimadzu). Separations were performed at 0.5 ml min^−1^ using a Force C18 column (50 × 2.1 mm, 3 µm, Thames Restek) fitted with a matching guard column (Force C18, 5 × 2.1 mm, 5 µm), and a delay column (50 × 2.1 mm, 5 µm, Thames Restek) was installed between the mobile phase mixer and the autosampler. Sample order was randomized throughout the batch with an injection volume of 10 µl, and the autosampler was held at 4 °C for the entire analysis. The elution programme is as follows: an initial hold at 30% mobile phase B (MPB, MeOH; MPA—10 mM ammonium acetate (aq)) for 0.51 min, then an increase to 60% MPB by 1.5 min, followed by another increase to 90% MPB at 5 min, a hold at 95% MPB from 6 to 7 min, followed by a 3-min re-equilibration period at initial conditions. A 90:10 (MeOH:H_2_O) needle wash was used to rinse the outside of the needle before and after sample aspiration. Three transitions were monitored for both PFNA (463.05 > 419.00, 463.05 > 219.25 and 463.05 > 169.30) and M9PFNA (472.00 > 427.15, 472.00 > 223.10 and 472.00 > 172.25). Refer to Supplementary Table 30 for the voltages and collision energies (optimized) used in each of the quadruples for each transition of PFNA and M9PFNA. Analysis of all extracts was accompanied by quality control samples (0, 1 and 10 µg l^−1^ in methanol, 1% ammonium hydroxide, 0.5% acetic acid) and mobile phase blanks to account for instrumental contamination and to mitigate carryover.

### LC–MS/MS (QQQ) metabolomics analysis

A deep-well plate containing 1,180 µl PFNA in mGAM was inoculated with 10 µl second-passage culture to create a final concentration of 20 µM PFNA (Fig. 4d,e and Extended Data Fig. 9). Samples were incubated at 37 °C for 24 h, after which 1 ml bacterial culture was transferred to a fresh tube and centrifuged at 4 °C and 3,202 × *g* for 3 min. The supernatant and pellet were collected and kept at −80 °C until extraction. Pellet samples and 100 µl of supernatant per sample were extracted with 800 µl of 1:1 methanol:water and vortexed; 200 µl chloroform was added to each sample and samples were incubated at −20 °C for 1 h for protein precipitation. Samples were then centrifuged at 4 °C and 3,202 × *g* for 2 min, and the water phase was transferred to LC–MS vials for analysis.

Amino acids and vitamins were quantified using liquid chromatography–tandem mass spectrometry as described previously^62^. On an Agilent 1290 Infinity II system, analytes were separated using hydrophilic interaction liquid chromatography with a Waters Acquity BEH Amide 1.7 µm, 2.1 mm × 100 mm column operated at 35 °C. A binary buffer system of buffer A (50% acetonitrile, 10 mM ammonium formate, 0.176% formic acid) and buffer B (95:5:5 acetonitrile:methanol:water, 10 mM ammonium formate, 0.176% formic acid) was used at a constant flow rate of 0.9 ml min^−1^ and the following gradient: 0 min: 85% B, 0.7 min: 85% B, 2.55 min: 5% B, 2.9 min: 5% B, 2.91 min: 85% B and 3.5 min: stop time. The Agilent triple quadrupole 6470 instrument with JetStream ion source (AJS-ESI) was used in dMRM mode with a cycle time of 320 ms. The source parameters were as follows: gas temperature, 325 °C; gas flow, 10 l min^−1^; nebulizer, 40 psi; sheath gas temperature, 350 °C; sheath gas flow, 11 l min^−1^; capillary (positive), 3,500 V; capillary (negative), 3,500 V; nozzle voltage (positive), 1,000 V; and nozzle voltage (negative), 1,000 V. The injection volume was 0.25 µl. A serially diluted external calibration standard, blanks and a pooled quality control sample were injected at regular intervals between samples. Data were analysed using MassHunter Workstation Quantitative Analysis for QQQ v10.1.

### LC–MS/MS data analysis

The Agilent MassHunter Qualitative Analysis 10.0 software was used to qualify the selected xenobiotic standards. Total ion chromatogram (TIC), extracted ion chromatogram (EIC) and EIC-fragment graphs were extracted for each compound. The Agilent MassHunter TOF Quantitative Analysis (version 10.1) or Agilent MassHunter QQQ Quantitative Analysis software (version 10.1) was used to quantify the xenobiotic compounds in each sample.

Calibration curves based on pure compound standards were used to estimate the concentrations of target compounds. Raw response values were used for concentration calculations for most analysis. Exceptions were the ‘Single strain-xenobiotic screen’, data represented in Extended Data Fig. 2i, low concentration experiment per EPA Draft Method 1633, PFAS-mix experiment and mouse faecal analysis, in which the ratio of the compound response to internal standard response was used. Data analysis was performed in RStudio Version 1.3.1093. The median of each sample group was compared with the median of the compound controls, and an appropriate reduction of more than 20% was chosen as cut-off, to ensure relevant reduction compared with the compound control distribution. In addition, statistical comparison was performed using *t*-test (two sided), and *P* values were false discovery rate (FDR) corrected (when a *t*-test was performed, the adjusted *P* values are given in the respective figures). An adjusted *P* value of less than 0.05 was considered significant.

In the ‘Single strain-xenobiotic screen’, bioaccumulation was defined as compound sequestration to at least 20% from the supernatant but not from the whole culture sample, whereas biodegradation was defined as both supernatant and whole culture sample showing more than 20% compound sequestration.

Concentration in samples extracted according to EPA Draft Method 1633 was calculated based on the ratio with the labelled IS, which was spiked into the sample at a known concentration of 100 ng l^−1^.

### PFAS–bacteria growth screens

Plates (Corning 3795) containing 50 μl mGAM with 2× PFAS (2% DMSO) concentration were prepared the evening before and placed into the anaerobic chamber overnight to ensure anaerobic conditions for inoculation (Fig. 1h and Extended Data Fig. 3b). On the day of the screen, each well was inoculated with 50 μl of a second-passage culture to reach a starting OD_600_ of 0.05. The plates were sealed with a gas-permeable membrane (Breath-Easy, Merck, catalogue number Z380059), which was additionally pierced with a syringe to prevent gas build-up. The plates were stacked without lids and incubated at 37 °C for 24 h in a stacker–incubator system (Biostack 4, Agilent BioTek) connected to a plate reader (Epoch 2, Agilent BioTek) to record the OD_600_ (ref. ^63^).

Growth curve analysis was performed in RStudio version 1.3.1093. First, for each growth curve, the minimum OD value was set to 0. Then, the raw area under the curve (AUC) was calculated for each well using ‘bayestestR’ package and area_under_curve() function. Further processing of growth curves was done by plate. AUC values were normalized by median AUC of all control wells (DMSO controls) on the respective plate to determine percentage growth inhibition.

### Conventional ultrathin-section TEM

On the day of the experiment, each tube containing 2× concentration of PFAS was inoculated 1:1 with a second-passage culture to reach a starting OD_600_ of 0.05 (Extended Data Fig. 4). Samples were incubated at 37 °C for 24 h, after which bacterial cultures were spun down and the supernatant was removed. The bacteria were then fixed with a half Karnovsky fixative as 2.5% glutaraldehyde and 2% paraformaldehyde in 0.1 M sodium cacodylate buffer (pH 7.4 with NaOH) for a few hours at room temperature. For conventional TEM^64^, the post-fixation was performed with a mixture of 1% osmium tetroxide and 1% potassium ferrocyanide in the cacodylate buffer. The bloc was stained with 5% aqueous uranyl acetate solution. The dehydration with a series of ethanol and the resin infiltration were completed for the plastic embedding in TAAB epoxy resin. After the polymerization at 65 °C for a few days, the ultrathin sections (∼60 nm) were cut using an ultramicrotome (Leica EM UCT/UC7/Artos-3D), mounted on formvar-carbon films on EM copper grids and stained with lead citrate. The bacterial ultrastructure was observed using a FEI Talos F200C 200 kV transmission electron microscope (Thermo Fisher Scientific) with a Ceta-16M CMOS-based camera (4k × 4k pixels under a 16-bit dynamic range) and a JEM-1400 Flash TMP (JEOL) with TVIPS TemCam-XF416 CMOS (Tietz Video and Image Processing Systems) as described previously^65^.

### Automated TEM image sectioning and analysis

Electron microscopy images were converted to an 8-bit format using FIJI^66^ (Extended Data Fig. 5). Only images with the same magnification level were analysed. The segmentation and quantification of these images were performed using CellProfiler 4.2.6 (Stirling, DR, BMC Bioinformatics, 2021^67^). Individual cells were segmented as primary objects based on their intensity and size after the application of a Gaussian blur filter. Within each cell, condensates were also segmented based on intensity and size. Mean pixel intensity and number of condensates were quantified for each cell and their corresponding condensates, and the results were exported as CSV files. Cells touching the edges of the images were not considered for this quantification. Images showing the segmented areas were saved as TIFF files. All sectioned cells and aggregates were manually checked, and wrong sectioned cells and cells that were identified in duplicate were excluded from further analysis.

### Cryogenic FIB-SIMS imaging

Cryogenic FIB-SIMS imaging^68^ was performed on a focused ion beam scanning electron microscope (Zeiss Crossbeam 550) equipped with a time-of-flight mass spectrometer (Tofwerk; Fig. 3 and Extended Data Fig. 6). During FIB-SIMS imaging, the sample is scanned by a gallium focused ion beam, which ablates material at each pixel. The secondary ions resulting from the interaction of the ion beam and sample are extracted at each scanning position and are analysed by time-of-flight secondary electron mass spectrometry. The method hence allows a pixel-by-pixel visualization of the sample’s chemical composition, in which the colour scale represents the ion count. The simultaneous detection of secondary electrons yields spatial images of the sample (FIB images, Fig. 3a). As material is continuously removed during the imaging process, repeated scanning of a given sample area provides three-dimensional information regarding both spatial features and chemical composition of the sample, thus creating a volume with a mass spectrum for each voxel. This not only allows this method to show the lateral localization of elements of interest, but also provides depth information: each two-dimensional image resulting from a full scan of the observed area can be considered as a slice of the sample (frame) within a *Z*-stack (Fig. 3) while the distribution along the *Z-*axis can be extracted as *X*–*Z* slices. To retain a near-native state of the sample, *E. coli ∆tolC* cells were plunge-frozen in liquid ethane using a Vitrobot Mark IV (Thermo Fisher) after applying 2.5 µl of sample to a cryo-EM grid (Quantifoil Cu/Rh R3.5/1, UltrAuFoil R 1.2/1.3) as described previously^69^.

### Proteomics analysis

A deep-well plate containing 1,180 µl PFNA in mGAM was inoculated with 10 µl second-passage culture to create a final concentration of 20 µM PFNA (Fig. 4a and Extended Data Fig. 8). Samples were incubated at 37 °C for 24 h, after which bacterial cultures were spun down and the supernatant was removed.

The bacterial cell pellets were lysed in 100 mM triethyl ammonium bicarbonate containing 0.1% RapiGest surfactant by sonication on ice (50J ×5, 30% amplitude) after denaturing by two rounds of incubation at 80 °C for 10 min with intermittent cooling. The solubilized protein content was estimated using Pierce 660 nm assay as per the manufacturer’s instructions. Then, 50 µg of protein from each sample was reduced with 4 mM dithiothreitol, alkylated with 14 mM iodoacetamide and further digested with trypsin at 1:50 protease to protein ratio for 16 h at 37 °C. The peptide content was estimated at 1:10 dilution using Pierce quantitative colorimetric assay as per the manufacturer’s instructions. Equal amounts of peptides from respective bacterial samples were labelled with TMT in a randomized fashion to ensure that the reporter ion isotopic distribution is spread across the replicates following the manufacturer’s guidelines (Thermo Fisher Scientific). An equal volume of the respective tandem mass tag (TMT)-labelled peptides was pooled together and desalted to remove unbound labels. The desalted peptides were further subjected to fractionation based on their reverse-phase chromatographic properties under basic pH using an analytical high-performance liquid chromatography (HPLC). The 12 concatenated fractions collected were vacuum dried, resuspended in 0.1% trifluoroacetic acid containing 3% acetonitrile and taken for LC–MS/MS analysis.

LC–MS/MS analysis was carried out in RTS-SPS-MS3 mode for TMT reporter ion quantification. An equal amount of predetermined sample load from each of the 12 fractions was analysed using an Orbitrap Eclipse mass spectrometer with an Ultimate 3000 RSLC nano chromatography system coupled in-line. The peptides were loaded onto the trapping column (Thermo Fisher Scientific, PepMap100, C18, 300 μm × 5 mm), using partial loop injection, washed for 3 min at a flow rate of 15 μl min^−1^ with 0.1% formic acid (FA). The peptides were resolved on an analytical column (Easy-Spray C18 75 µm × 500 mm; 2 µm particle size) at a flow rate of 300 nl min^−1^ using a gradient in which the percentage of B is raised from 3–25 over 140 min and then to 40% for an additional 13 min. The column was washed in 90% B for 12 min and re-equilibrated in 3% B for 15 min before next injection. Subsequently, 0.1% FA in water was used as mobile phase A and 0.1% FA in 80% acetonitrile was used as mobile phase B. Data were acquired using three field asymmetric ion mobility spectrometry (FAIMS) compensation voltages (−45 V, −60 V and −75 V). For each FAIMS experiment with a maximum cycle time of 2 s, mass spectrum acquisition was carried out in RTS-SPS-MS3 mode. In this mode, full-scan MS was acquired in the mass range of 415–1,500 m/z at 120,000 resolution with a maximum ion injection time of 30 ms (AGC target 2e5 ions). Precursors selected for MS/MS were isolated using an isolation width of 0.7 m/z and fragmented by HCD using collision energy of 32%. An MS/MS scan was performed on the ion trap at a turbo scan rate (AGC 5e4 ions) with a maximum ion injection time of 22 ms. To avoid repeated selection of peptides for MS/MS, the programme used a 40-s dynamic exclusion window. Real-time search parameters were set as follows using respective fasta databases: Uniprot *B. uniformis* database, UP000014212 (downloaded on 21 June 2023, contains 3,957 sequences); Uniprot *O. splanchnicus* database, UP000006657 (downloaded on 21 June 2023, contains 3,479 sequences); and Uniprot *E. coli* K12 database, UP000000625 (downloaded on 21 June 2023, contains 4,362 sequences). Protease: trypsin, static modifications: cysteine carbamidomethylation (+57.0215) and TMT (+229.163) or TMTPro (+304.207) on lysine and N-terminus, variable modification: methionine oxidation (+15.99491). One missed cleavage was allowed, and FDR filtering was enabled. Only 5 peptides per protein were allowed per basic reverse-phase fraction (using the close-out option), and the maximum search time for RTS was set to 35 ms. Ten of the most abundant peptide fragments were selected for SPS-MS3, and the MS3 spectrum was acquired at 120,000 resolution on the mass range 100–500 m/z with an AGC target of 500% and a maximum injection time of 246 ms.

Data were processed using Proteome Discoverer v3.0 using the protein databases mentioned above. The spectrum identification was performed with the following parameters: MS accuracy, 10 ppm; MS/MS accuracy, 0.6 Da for spectra acquired in an ion trap mass analyser; missed cleavage, up to 2; fixed modifications, carbamidomethylation of cysteine and TMT/TMTpro on lysine and on peptide N-terminus; and variable modifications, oxidation of methionine. An intensity-based rescoring of PSMs identified in SequestHT node was performed by Inferys before false discovery rate estimation using Percolator. Only rank 1 peptide identifications with high confidence (FDR < 1%) were accepted for identification and quantification reporting. Quantification was carried out on the S/N values of reporter ions for the identified peptides that are unique to the proteins. The protein abundance was enumerated by summing the abundances of the connected peptide groups matching to respective proteins. Data normalization was carried out using the TMT channel with the highest total protein abundance as the reference channel. The normalized abundance values are reported for comparison across datasets.

### TPP

TPP was performed as previously described^36,70^ (Fig. 4b,c). In brief, cells were grown anaerobically at 37 °C for 48 h. Cells were then washed twice with anaerobic PBS. For the live cell experiments, cells were treated with eight different concentrations of anaerobic PFNA (twofold dilutions starting at 10 µM including DMSO control) for 30 min at 37 °C in the anaerobic chamber. Aliquots of treated cells were then incubated in 10 different temperatures for 3 min. Cells were resuspended in lysis buffer (50 µg ml^−1^ lysozyme, 1 mM MgCl_2_, complete protease inhibitors, 0.25 U µl^−1^ benzonase in PBS) with 0.8% NP-40 and lysed with 5 freeze–thaw cycles. Aggregates were then removed and soluble proteins were prepared for mass spectrometry. For the lysate experiments, cells were lysed as above but without the presence of NP-40. NP-40 was added after the heat treatment before the aggregate removal.

Proteins were digested as previously described^71,72^. Eluted peptides were labelled with TMT16plex, pooled (all treatments of each two adjacent temperatures per experiment) and pre-fractionated into six fractions under high-pH conditions. Samples were then analysed with liquid chromatography coupled to tandem mass spectrometry, as previously described^70^.

All raw files were converted to mzmL format using MSConvert from Proteowizard, using peak picking from the vendor algorithm. Files were then searched using MSFragger v3.7 (ref. ^73^) in Fragpipe v19.0 against the Uniprot proteome UP000000625 containing common contaminants and reversed sequences.

Data analysis was performed using R. Only proteins with at least two identified peptides were kept for the analysis, and outlier conditions were removed altogether. For the correlation analysis, per experiment, protein intensities across all concentrations within each temperature were normalized using variance stabilization normalization^74^ and the cumulative fold change across temperatures and concentrations per protein was calculated. For the hit calling, the data were preprocessed using the TPP package^49^ and analysed using the TPP2D package^75^ with an alpha of 0.1.

### Adaptive laboratory evolution

*B. uniformis*, *B. thetaiotamicron*, *O. splanchnicus*, *P. merdae* and *E. coli* BW25113 ∆*tolC* were evolved for 20 days in 500 μM PFHpA, 500 μM PFOA, 250 μM PFNA and 125 μM PFDA (Extended Data Fig. 7). DMSO was used as a control. For each PFAS compound 4 and for the DMSO controls 8 replicate lineages were evolved in parallel. Subsequently, 2-ml deep-well stock plates containing a 100× stock of each PFAS in DMSO were prepared before the experiment and stored at −80 °C until use. On day 0, each well was inoculated with a second-passage culture to reach a starting OD_600_ of 0.05. On each of the following days, 50 μl of grown culture was transferred to a fresh compound plate containing PFAS–DMSO in mGAM. Every 5 days, the growth of the strains in the presence of PFAS was measured by transferring 100 μl of starting culture to a clear-bottom plate, measuring and analysing it as described in the section ‘PFAS–Bacteria Growth Screens’. On day 20, glycerol stocks were prepared from each lineage and stored at −80 °C.

#### Genome sequencing

Genomic DNA of *B. uniformis* after evolving in PFNA, PFOA and DMSO control was extracted with the QIAamp PowerFecal Pro DNA kit (Qiagen; 51804) and quantified using Qubit 1X dsDNA HS Assay Kits (Q33230). On average, 74.38 ng µl^−1^ of DNA was obtained and sequenced by Illumina NovaSeq 6000 with a library insert size of 350 bp, resulting in 6.50 to 9.95 million paired reads (mean = 8.68 million) with a length of 150 bp. Sequencing reads were then filtered with fastp (v 0.32.2)^76^ using default parameters. We called the variants in evolved strains using snippy pipeline (https://github.com/tseemann/snippy). Filtered reads from PFNA-, PFOA- and DMSO-evolved populations were aligned to the reference genome with BWA, and variants were called by freebayes^77^ using parameters ‘–mincov 10–basequal 30–fbopt '-C 3 -p 1 -F 0.01'’ defined through snippy. Afterwards, those variants appearing in both PFNA–PFOA and DMSO control samples were removed.

### Mouse experiments

Animal experiments were approved by the local authorities (Regierungspräsidium Tübingen, H 02/20 G) (Fig. 5 and Extended Data Fig. 10). GF C57BL/6J mice were bred in house (Gnotobiotic Mouse Facility). Mice were housed under GF conditions in flexible film isolators (Zoonlab) and transferred to the Isocage P system (Tecniplast) to perform the experiments. The housing conditions were 12:12-h light–dark cycles; temperature, 22 °C ± 2 °C; and humidity, 50–56%. Mice were supplied with autoclaved drinking water and γ-irradiated maintenance chow for mice (Altromin) ad libitum. Female and male mice between 5 and 6 weeks old were randomly assigned to experimental groups. Mice were kept in groups of three per cage during the experiment. All animals were scored daily for their health status.

#### Preparation and inoculation of the Com20 and high- versus low-accumulating bacterial community

The Com20 and high- and low-accumulating communities (Supplementary Table 6) were prepared under anaerobic conditions (Coy Laboratory Products; 2% H_2_, 12% CO_2_ and the remainder was N_2_). Consumables, glassware and media were pre-reduced at least 2 days before inoculation of bacteria. Each strain was grown in monoculture overnight in 5 ml of their respective growth medium at 37 °C. The next day, bacteria were subcultured (1:100) in 5 ml fresh medium and incubated for 16 h at 37 °C, except *Eggerthella lenta*, which was grown for 2 days. OD at 578 nm was determined, and bacteria were mixed together in equal ratios to a total OD of 0.5 in a final volume of 10 ml. After 2.5 ml of 50% glycerol (with a few crystals of palladium black (Sigma-Aldrich)) was added, 200-µl aliquots were prepared in glass vials (2 ml; Supelco, ref. 29056-U) and directly frozen at −80 °C. Frozen vials were used within 3 months.

Inoculation of GF mice was performed according to a previous study^55^. In short, cages were transferred to an ISOcage Biosafety Station (IBS) (Tecniplast) through a 2% Virkon S disinfectant solution (Lanxess) dipping bath. Glycerol stocks of the frozen communities (one per mouse) were kept on dry ice before being thawed during transfer into the IBS. Mixtures were used directly after thawing with a minimal exposure time to oxygen of maximum 3 min. Mice were inoculated by oral gavage (50 µl) and inoculation was repeated after 48 h using the same protocol. The GF control group was left untreated. The IBS was sterilized with 3% perchloracetic acid (Wofasteril, Kesla Hygiene AG).

#### Faecal sample collection

Mice were orally gavaged with PFNA (10 mg kg^−1^ or 0.1 mg kg^−1^ in 25% DMSO) in a volume of 50 µl 10 days after the second inoculation with Com20, or high- or low-accumulating communities. The human equivalent dose of 0.1 mg kg^−1^ is 8.1 µg kg^−1^ (ref. ^78^), which amounts to 486 µg PFNA for a 60-kg person. Given the EU drinking water limit for individual PFAS at 0.1 µg l^−1^ and an estimated daily water intake of 4 l, this dose corresponds to approximately 3 years of exposure.

Fresh faecal samples were collected before treatment and 3 h, 1 day and 2 days after treatment in sterile 1.5-ml Eppendorf tubes and immediately frozen at −80 °C. On day 3 after treatment, mice were euthanized using CO_2_ and cervical dislocation and dissected, and intestinal contents were taken from the colon and the small intestine.

#### 16S rRNA gene sequencing of faecal samples

DNA extraction from faecal samples was performed in house using a MagMAX Microbiome Ultra Nucleic Acid Isolation Kit in bead tubes (Thermo Fisher Scientific, A42358) and the KingFisher Flex (MAN0018071) according to the manufacturer’s instructions. Subsequently, DNA integrity was verified using agarose gel electrophoresis and the DNA concentration was determined using a Qubit dsDNA BR assay kit (Thermo Fisher Scientific, Q32853) in combination with the Varioskan LUX plate reader (Thermo Fisher Scientific).

A two-step PCR protocol was used to prepare 16S amplicons. A single amplicon was generated using V4 primer pairs (515F/806R) for the first mouse experiment (10 mg kg^−1^ PFNA, Com20 versus GF) and V4 and V5 primer pairs (515F/926R) for the second mouse experiment (10 mg kg^−1^ PFNA, high- versus low-accumulating community). In the first step PCR, primers with overhang adapter sequences were added; afterwards, in the second PCR, Illumina sequencing adaptors and dual‐index barcodes were added to the amplicon target for pooling. Pooled samples are sequenced on an Illumina MiSeq instrument using a 2 × 250 PE protocol at the Genomics Core Facility (EMBL Heidelberg).

In total, 1,086,628 pair-end reads for the first mouse experiment and 6,531,459 for the second mouse experiment (an average of 10,061 pairs per sample in the first experiment and 56,306 pairs per sample in the second experiment) with 250 bp were generated. Raw sequencing reads were truncated and filtered using the DADA2 pipeline (v1.26.0)^79^ with the following parameters: ‘truncLen=c(230,210), maxN=0, maxEE=c(2,2), truncQ=2, rm.phix=TRUE’. Afterwards, error rates were learned from filtered reads, and corrected reads were merged as ASVs. A self-defined reference was used for alignments of ASVs and calculation of relative abundances.

### Data analysis and replicates

All data analysis was performed using open-source packages accessed from RStudio (version 1.3.1093). The Agilent MassHunter TOF Quantitative Analysis (version 10.1) or Agilent MassHunter QQQ Quantitative Analysis software (version 10.1) was used to quantify the xenobiotic compounds in each sample. All *t*-tests are two sided. All *P* values are FDR corrected. Biological replicates refer to different inoculation cultures, while technical replicates refer to experiments starting with the same inoculation culture. None of the data points correspond to repeated measurements of the same sample. Fold changes refer to the ratio of medians.

No randomization was used in microbiological experiments. In animal experiments, female and male mice were housed in separate cages, and subsequently, cages were randomly assigned to experimental groups. Each experimental group included mice from both sexes.

Data collection and analysis were not performed blind to the conditions of the experiments. No statistical methods were used to predetermine sample sizes. Sample sizes were chosen on previous experience and according to those reported in previous publications^36,56^.

### Reporting summary

Further information on research design is available in the Nature Portfolio Reporting Summary linked to this article.

## Supplementary information

Supplementary Information Supplementary Figs. 1 and 2.

Reporting Summary

Supplementary Tables Supplementary Tables 1–48 containing source data for Figs. 1, 2, 4 and 5 and Extended Data Figs. 1, 2, 3, 5, 7, 8, 9 and 10.

Supplementary Data Additional images from cryogenic FIB-SIMS imaging.

Supplementary Video A tomogram for *B. uniformis* cells exposed to 250 µM PFNA.

## Data Availability

Data that support the findings of this study are included in Supplementary Information. TEM images are available at the EBI Bioimage Archive (S-BIAD1892). The raw mass spectrometry data are available at the EBI MetaboLights repository (MTBLS9756, MTBLS9745, MTBLS10603, MTBLS10622, MTBLS10636). The raw proteomics and TPP data have been deposited in the ProteomeXchange Consortium via the PRIDE^80^ partner repository with dataset identifiers PXD050999 and PXD062206. Raw sequencing reads of the evolved *B. uniformis* and 16S rRNA genotyping were uploaded to the European Nucleotide Archive (PRJEB72794 and PRJEB75767).

## Code availability

No custom algorithms were used for data analysis. The code used is available via GitHub at https://github.com/AnnaELindell/PFAS-paper.
