# Supplementary material for: Human gut bacteria bioaccumulate per- and polyfluoroalkyl substances
Source: Nat Microbiol. 2025 Jul 1;10(7):1630–47. doi: 10.1038/s41564-025-02032-5 (PMC12222025; doi:10.1038/s41564-025-02032-5)
Supplement: Supplementary file 1 — Supplementary Figs. 1 and 2. [file 41564_2025_2032_MOESM1_ESM.pdf]

nature microbiology

Supplementary information

https://doi.org/10.1038/s41564-025-02032-5

Human gut bacteria bioaccumulate per- and polyfluoroalkyl substances

In the format provided by the authors and unedited

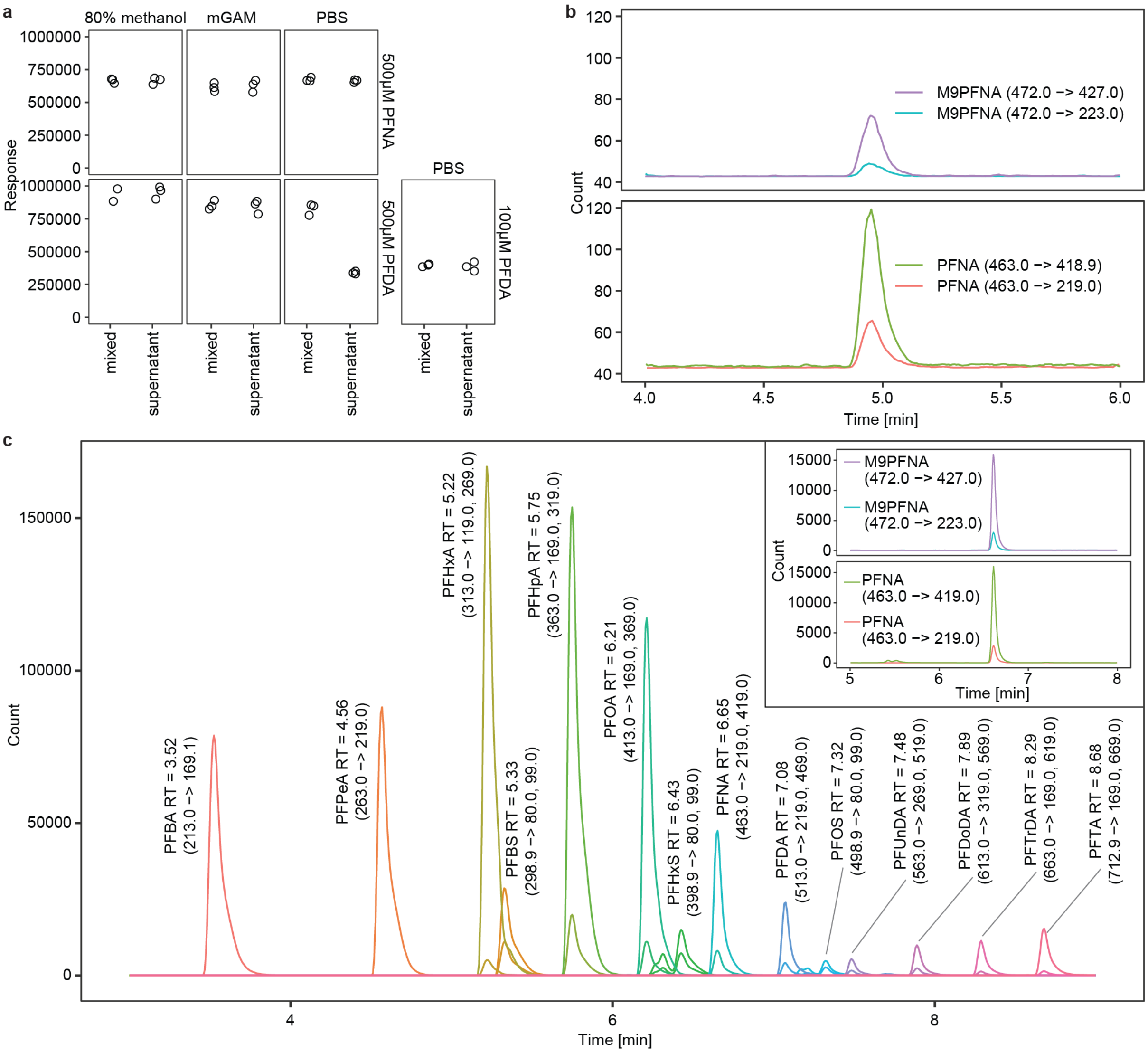

Supplementary Figure 1. a. Solubility of PFNA and PFDA in 80% methanol (1% DMSO), mGAM (1% DMSO) and PBS (1% DMSO). PFNA was soluble in all conditions up to 500 μM, while PFDA was soluble up to 500 μM in 80% methanol and mGAM, and up to 100 μM in PBS. n = 3 technical replicates (SI Table 45). **b.** PFNA and 13C-labelled PFNA chromatograms for 10 min reverse-phase LC method used with QQQ for mouse fecal sample analysis Fig. 5. **c.** Chromatograms for 14 PFAS compounds and their 13C labelled internal standards using the EPA Draft Method 1633 (Fig. 2d,f).

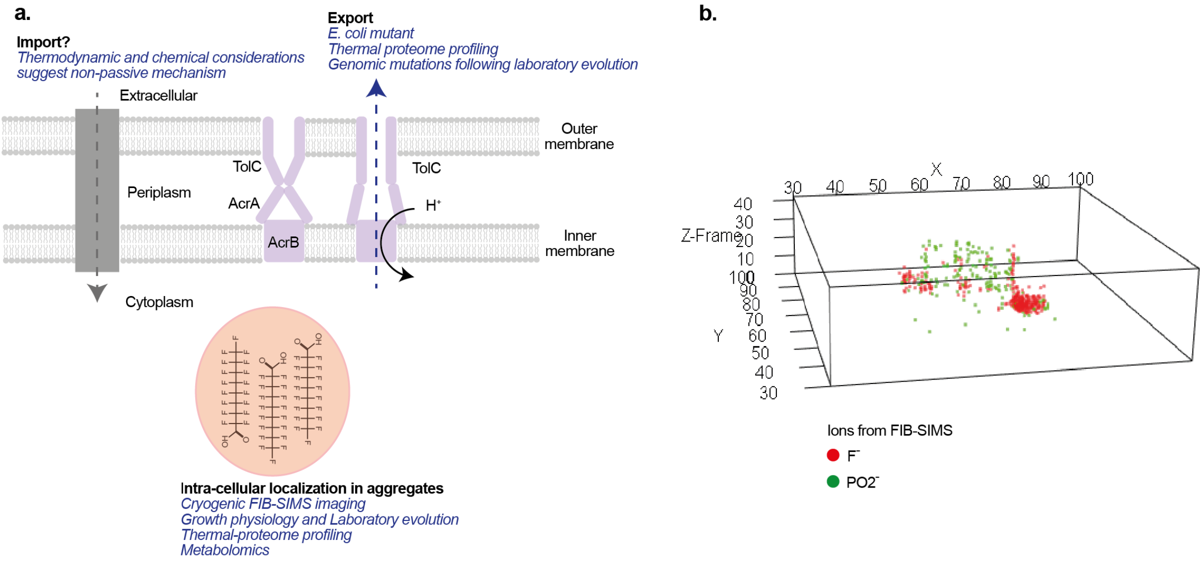

Supplementary Figure 2. Mechanistic insights into PFAS bioaccumulation by gut bacteria. a. The bioaccumulation involves three key stages: import, intra-cellular retention, and export. Our data provides mechanistic insights into the latter two steps. **b.** 3D rendering of the data shown in Figure 3. This data shows that the bioaccumulated PFAS molecules aggregate in dense clusters. Distribution of PO2 ions is shown as a control for visualization.
