# Supplementary figures and images for: Human gut bacteria bioaccumulate per- and polyfluoroalkyl substances
Source: Nat Microbiol. 2025 Jul 1;10(7):1630–47. doi: 10.1038/s41564-025-02032-5 (PMC12222025; doi:10.1038/s41564-025-02032-5)

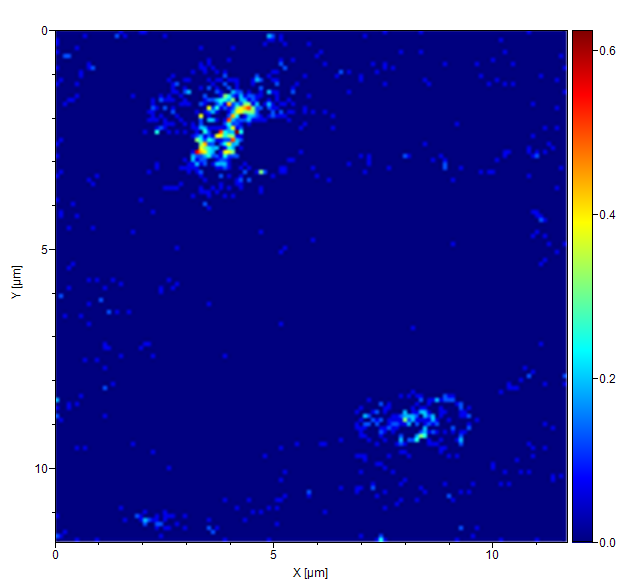

Supplement: Supplementary file 4 — Additional images from cryogenic FIB-SIMS imaging. [file 41564_2025_2032_MOESM4_ESM.zip › 20240507_Ecoli_PFNA_cell8.png]

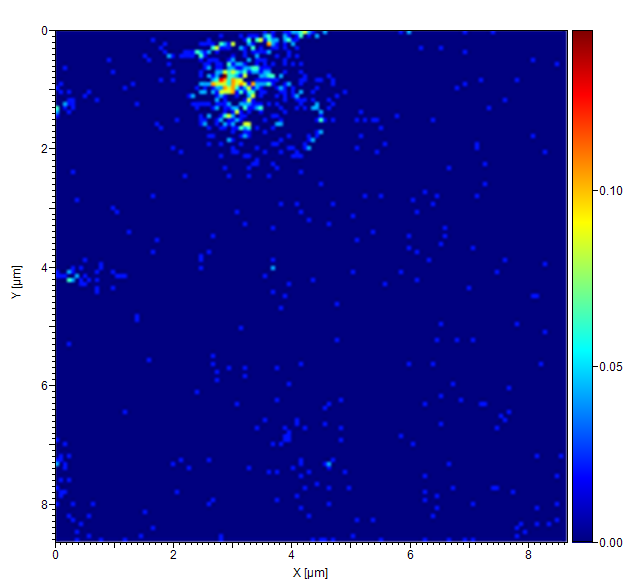

Supplement: Supplementary file 4 — Additional images from cryogenic FIB-SIMS imaging. [file 41564_2025_2032_MOESM4_ESM.zip › 20240507_Ecoli_PFNA_cell9.png]

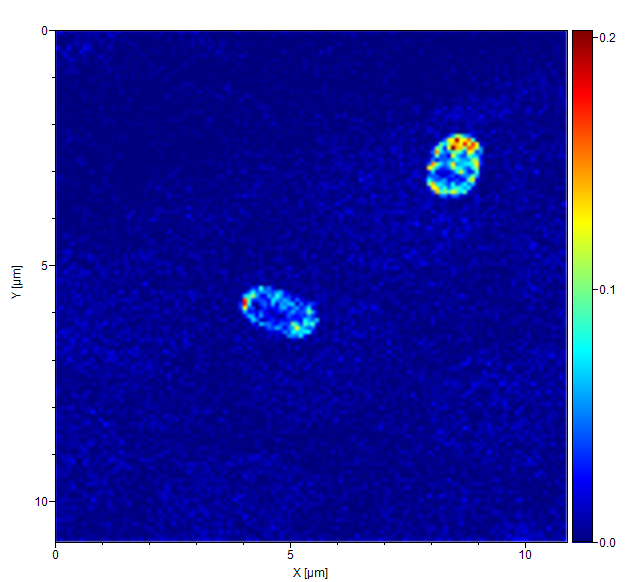

Supplement: Supplementary file 4 — Additional images from cryogenic FIB-SIMS imaging. [file 41564_2025_2032_MOESM4_ESM.zip › 20240507_Ecoli_PFNA_cell1.png]

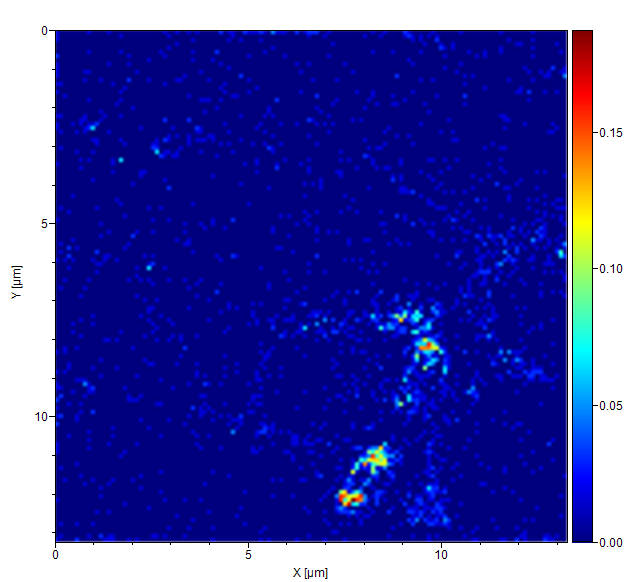

Supplement: Supplementary file 4 — Additional images from cryogenic FIB-SIMS imaging. [file 41564_2025_2032_MOESM4_ESM.zip › 20240507_Ecoli_PFNA_cell2.png]

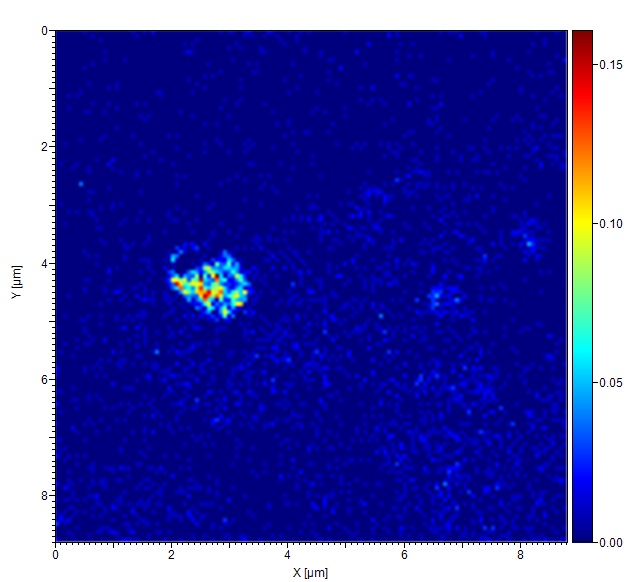

Supplement: Supplementary file 4 — Additional images from cryogenic FIB-SIMS imaging. [file 41564_2025_2032_MOESM4_ESM.zip › 20240507_Ecoli_PFNA_cell3.png]

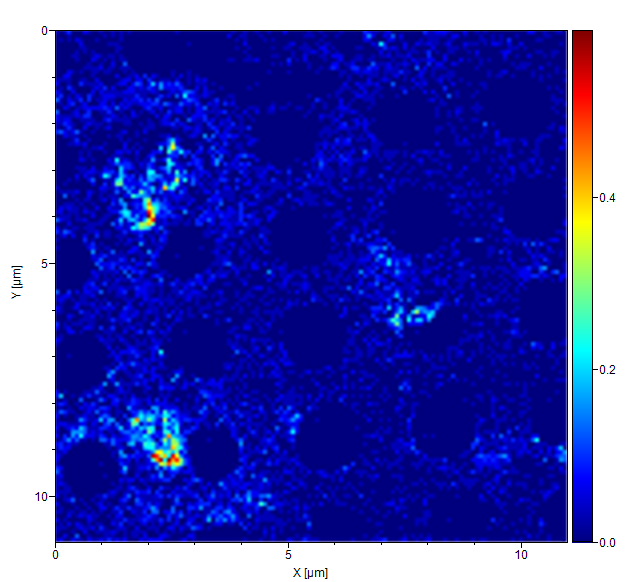

Supplement: Supplementary file 4 — Additional images from cryogenic FIB-SIMS imaging. [file 41564_2025_2032_MOESM4_ESM.zip › 20240507_Ecoli_PFNA_cell4.png]

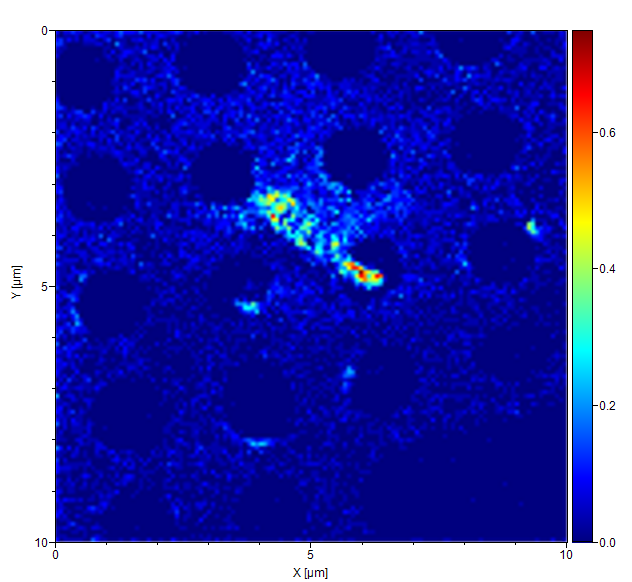

Supplement: Supplementary file 4 — Additional images from cryogenic FIB-SIMS imaging. [file 41564_2025_2032_MOESM4_ESM.zip › 20240507_Ecoli_PFNA_cell5.png]

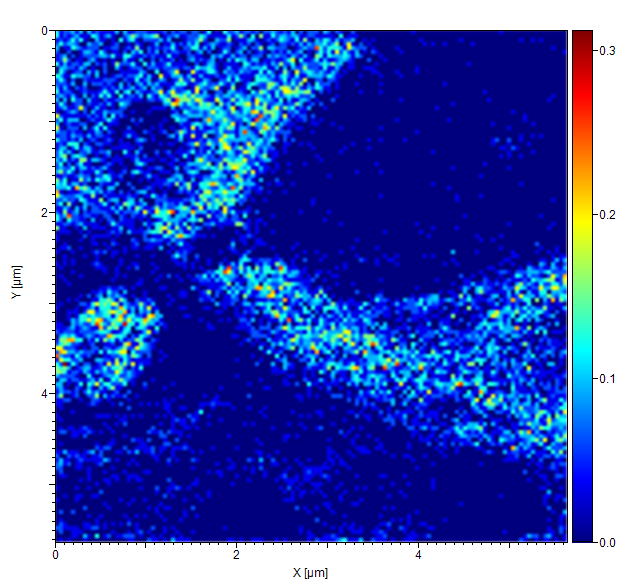

Supplement: Supplementary file 4 — Additional images from cryogenic FIB-SIMS imaging. [file 41564_2025_2032_MOESM4_ESM.zip › 20240507_Ecoli_PFNA_cell6.png]

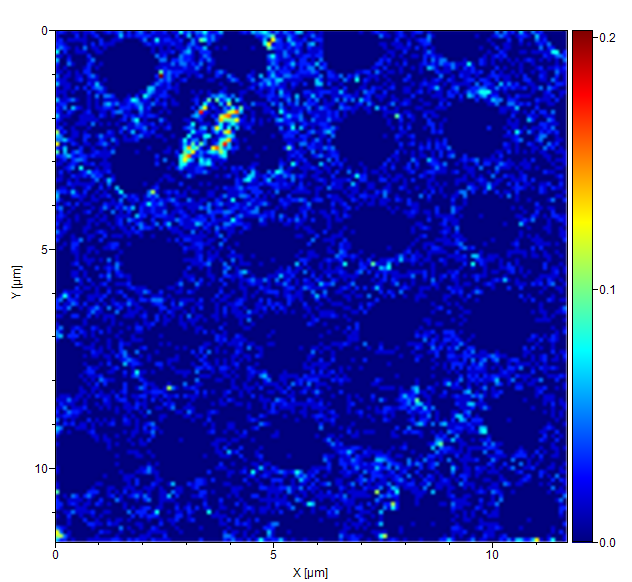

Supplement: Supplementary file 4 — Additional images from cryogenic FIB-SIMS imaging. [file 41564_2025_2032_MOESM4_ESM.zip › 20240507_Ecoli_PFNA_cell7.png]

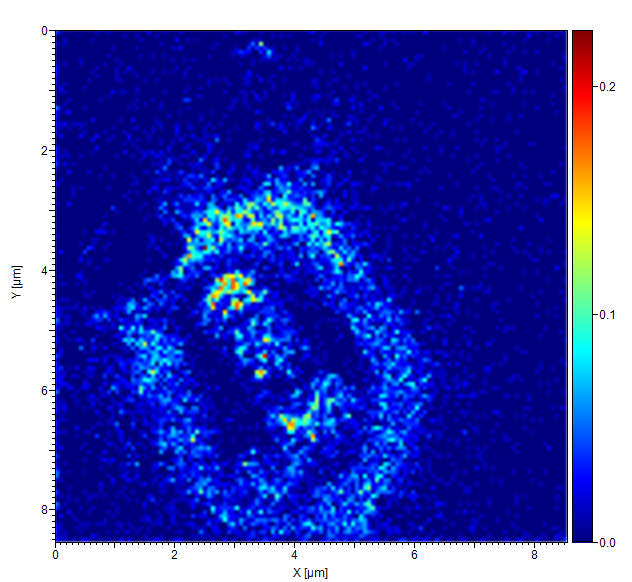

Supplement: Supplementary file 4 — Additional images from cryogenic FIB-SIMS imaging. [file 41564_2025_2032_MOESM4_ESM.zip › 20240507_Ecoli_PFNA_cell27.png]

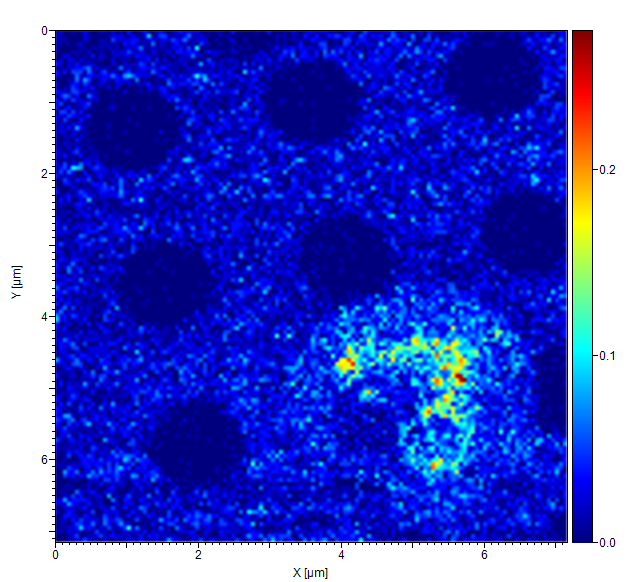

Supplement: Supplementary file 4 — Additional images from cryogenic FIB-SIMS imaging. [file 41564_2025_2032_MOESM4_ESM.zip › 20240507_Ecoli_PFNA_cell32.png]

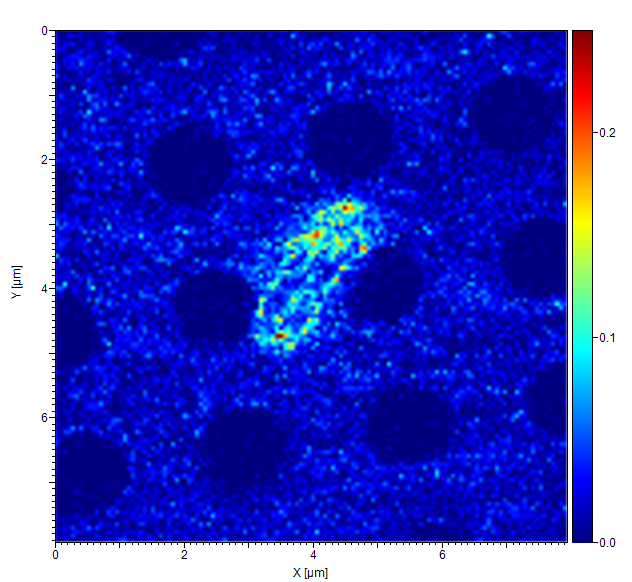

Supplement: Supplementary file 4 — Additional images from cryogenic FIB-SIMS imaging. [file 41564_2025_2032_MOESM4_ESM.zip › 20240507_Ecoli_PFNA_cell33.png]

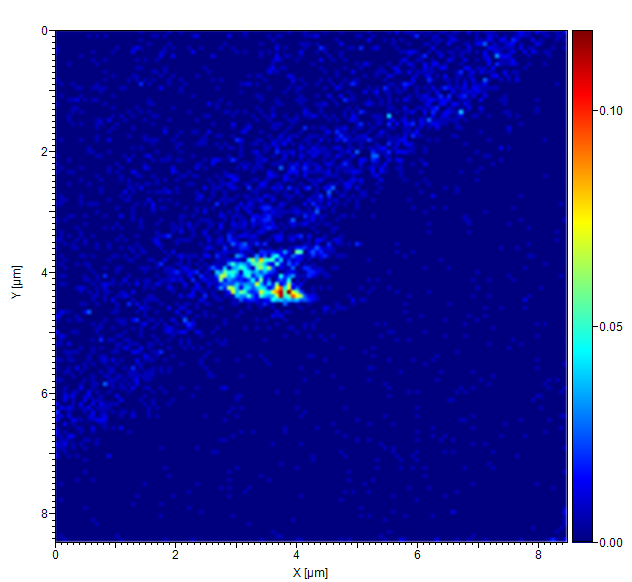

Supplement: Supplementary file 4 — Additional images from cryogenic FIB-SIMS imaging. [file 41564_2025_2032_MOESM4_ESM.zip › 20240507_Ecoli_PFNA_cell10.png]

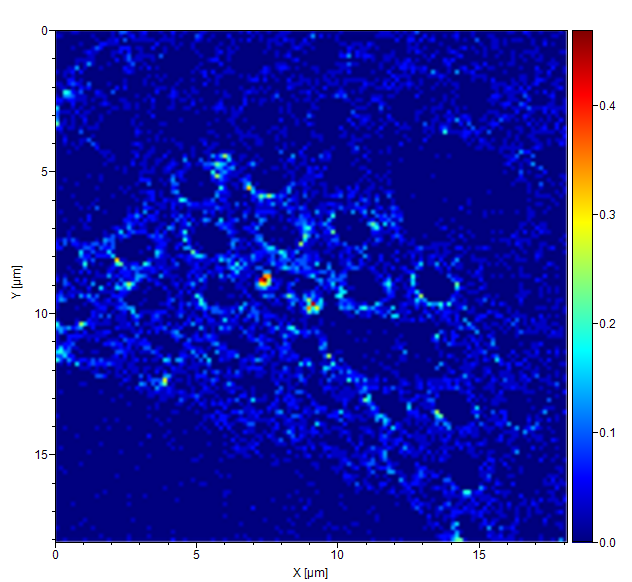

Supplement: Supplementary file 4 — Additional images from cryogenic FIB-SIMS imaging. [file 41564_2025_2032_MOESM4_ESM.zip › 20240507_Ecoli_PFNA_cell11.png]

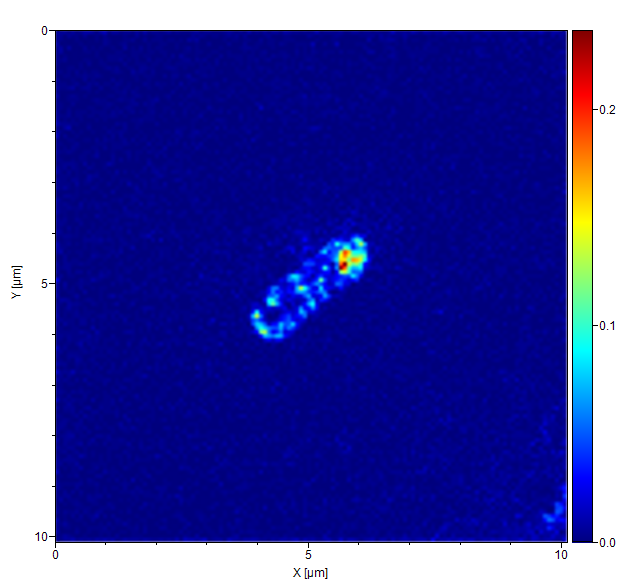

Supplement: Supplementary file 4 — Additional images from cryogenic FIB-SIMS imaging. [file 41564_2025_2032_MOESM4_ESM.zip › 20240507_Ecoli_PFNA_cell12.png]

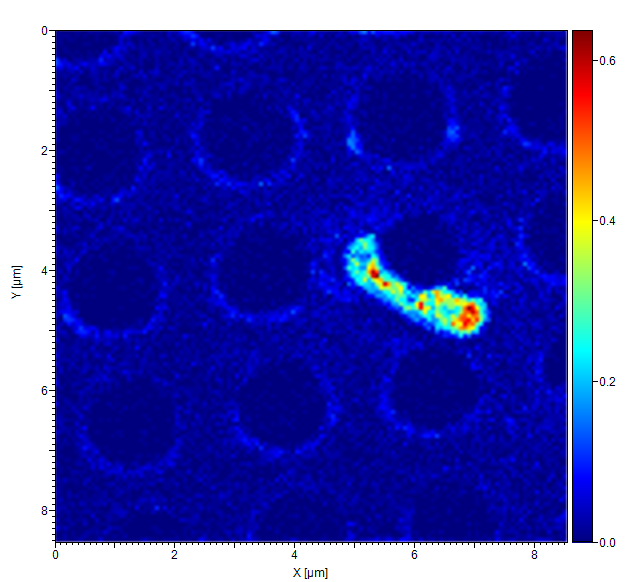

Supplement: Supplementary file 4 — Additional images from cryogenic FIB-SIMS imaging. [file 41564_2025_2032_MOESM4_ESM.zip › 20240507_Ecoli_PFNA_cell13.png]

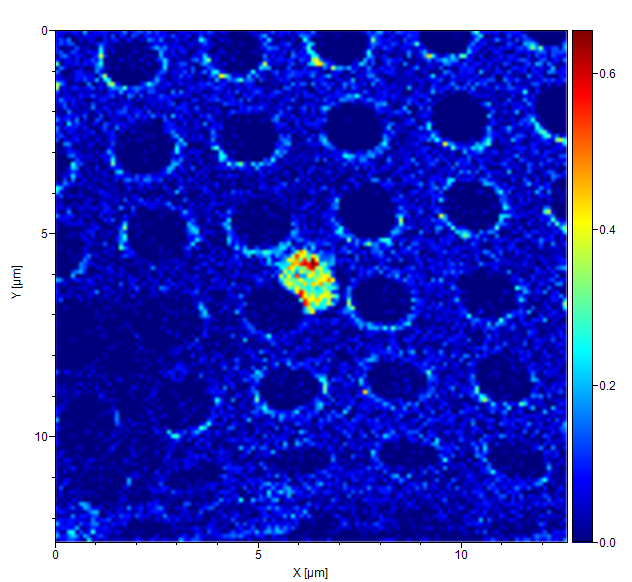

Supplement: Supplementary file 4 — Additional images from cryogenic FIB-SIMS imaging. [file 41564_2025_2032_MOESM4_ESM.zip › 20240507_Ecoli_PFNA_cell14.png]

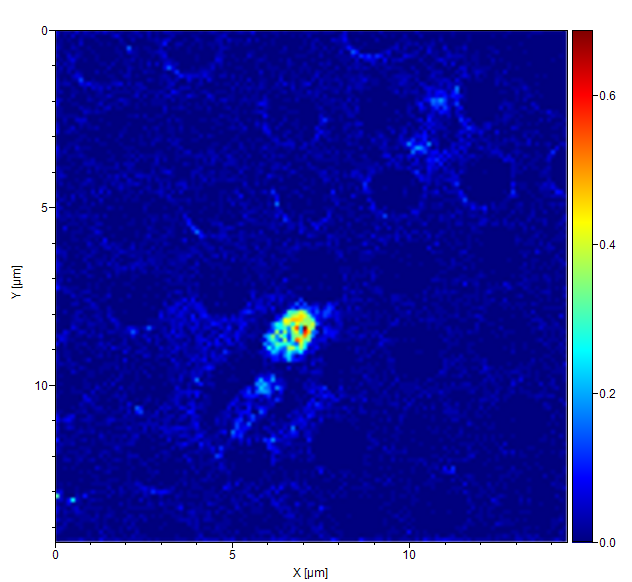

Supplement: Supplementary file 4 — Additional images from cryogenic FIB-SIMS imaging. [file 41564_2025_2032_MOESM4_ESM.zip › 20240507_Ecoli_PFNA_cell15.png]

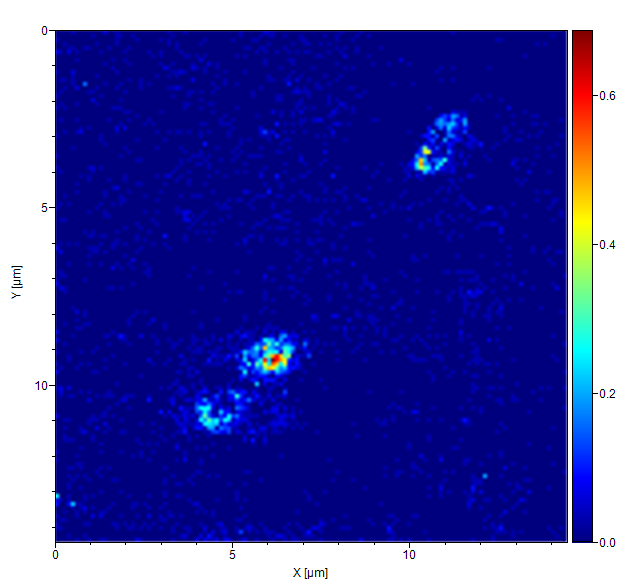

Supplement: Supplementary file 4 — Additional images from cryogenic FIB-SIMS imaging. [file 41564_2025_2032_MOESM4_ESM.zip › 20240507_Ecoli_PFNA_cell16.png]

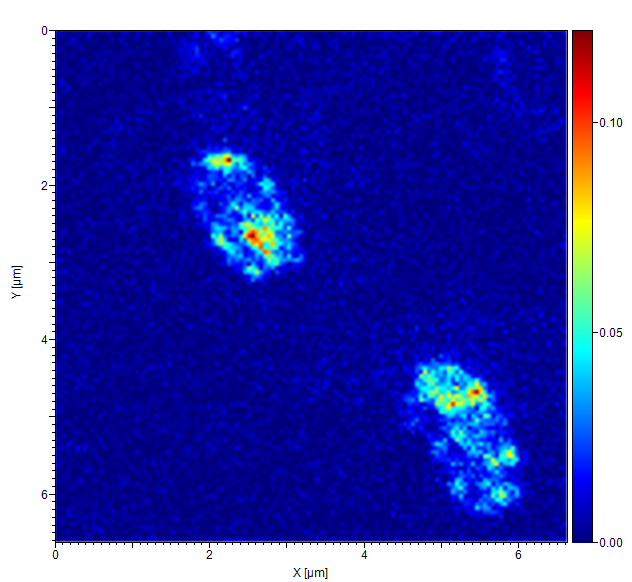

Supplement: Supplementary file 4 — Additional images from cryogenic FIB-SIMS imaging. [file 41564_2025_2032_MOESM4_ESM.zip › 20240507_Ecoli_PFNA_cell17.png]

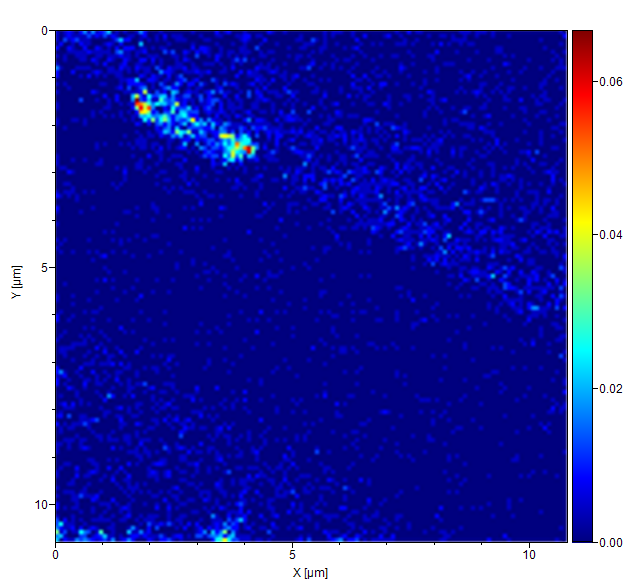

Supplement: Supplementary file 4 — Additional images from cryogenic FIB-SIMS imaging. [file 41564_2025_2032_MOESM4_ESM.zip › 20240507_Ecoli_PFNA_cell18.png]

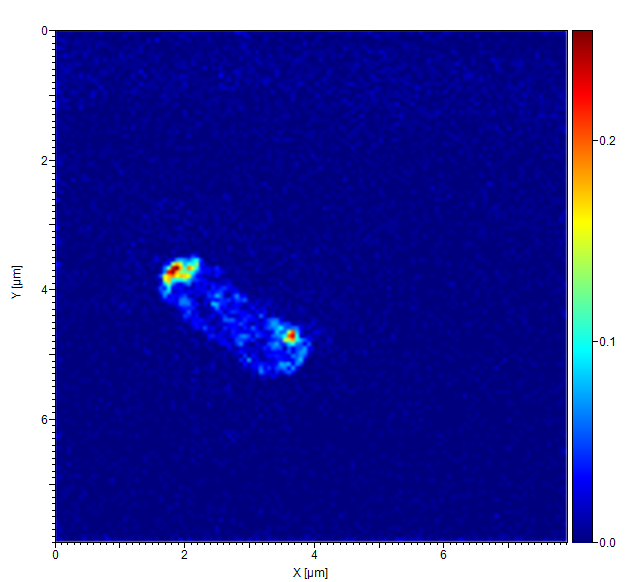

Supplement: Supplementary file 4 — Additional images from cryogenic FIB-SIMS imaging. [file 41564_2025_2032_MOESM4_ESM.zip › 20240507_Ecoli_PFNA_cell19.png]

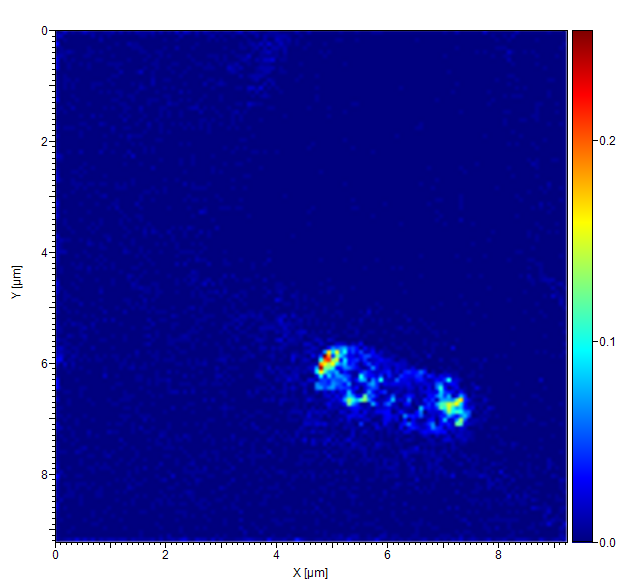

Supplement: Supplementary file 4 — Additional images from cryogenic FIB-SIMS imaging. [file 41564_2025_2032_MOESM4_ESM.zip › 20240507_Ecoli_PFNA_cell20.png]

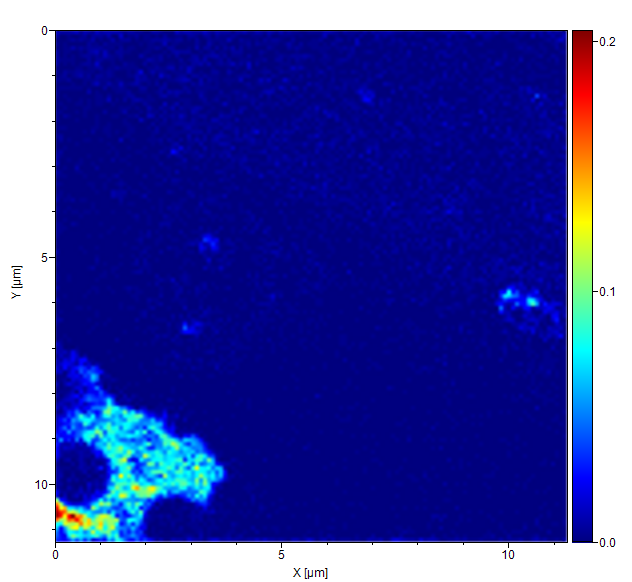

Supplement: Supplementary file 4 — Additional images from cryogenic FIB-SIMS imaging. [file 41564_2025_2032_MOESM4_ESM.zip › 20240507_Ecoli_PFNA_cell21.png]

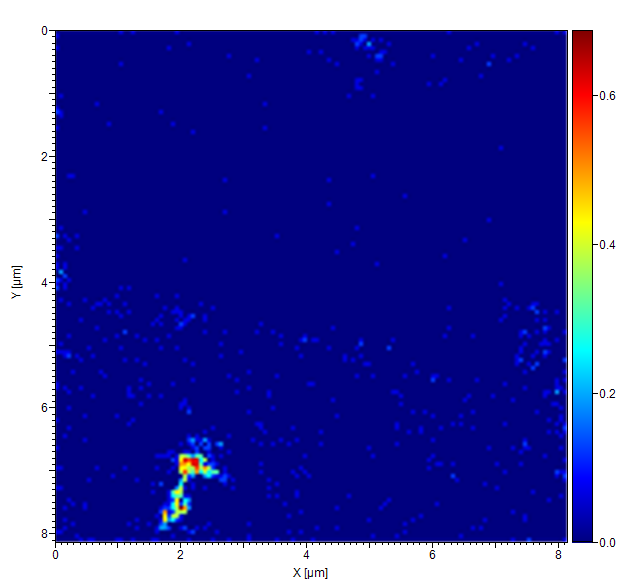

Supplement: Supplementary file 4 — Additional images from cryogenic FIB-SIMS imaging. [file 41564_2025_2032_MOESM4_ESM.zip › 20240507_Ecoli_PFNA_cell22.png]

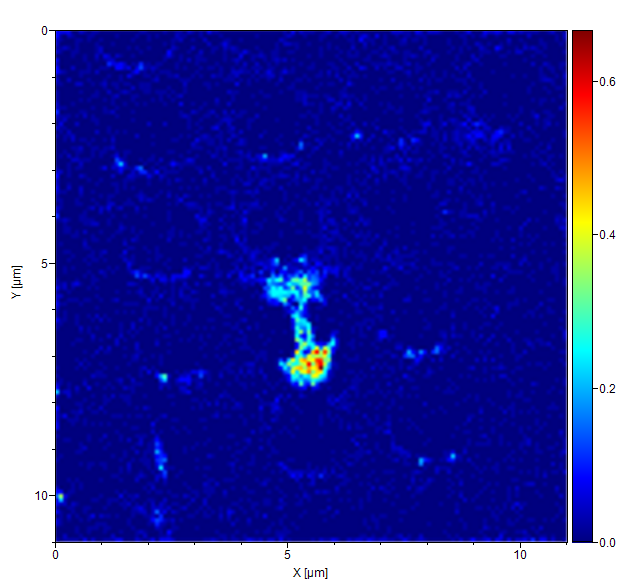

Supplement: Supplementary file 4 — Additional images from cryogenic FIB-SIMS imaging. [file 41564_2025_2032_MOESM4_ESM.zip › 20240507_Ecoli_PFNA_cell23.png]

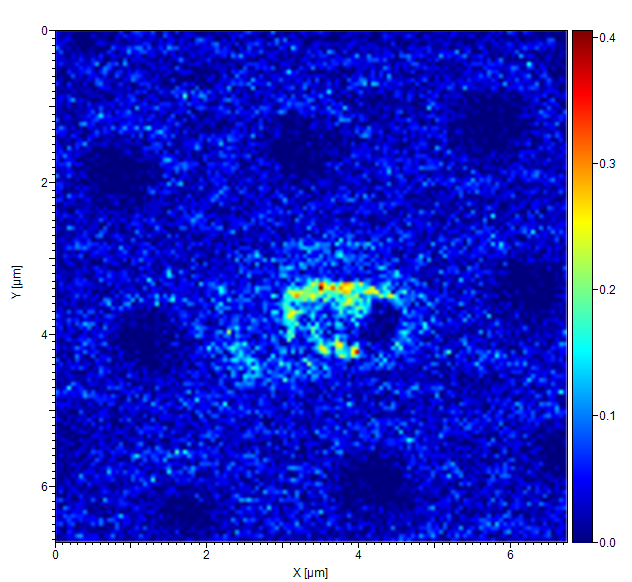

Supplement: Supplementary file 4 — Additional images from cryogenic FIB-SIMS imaging. [file 41564_2025_2032_MOESM4_ESM.zip › 20240507_Ecoli_PFNA_cell24.png]

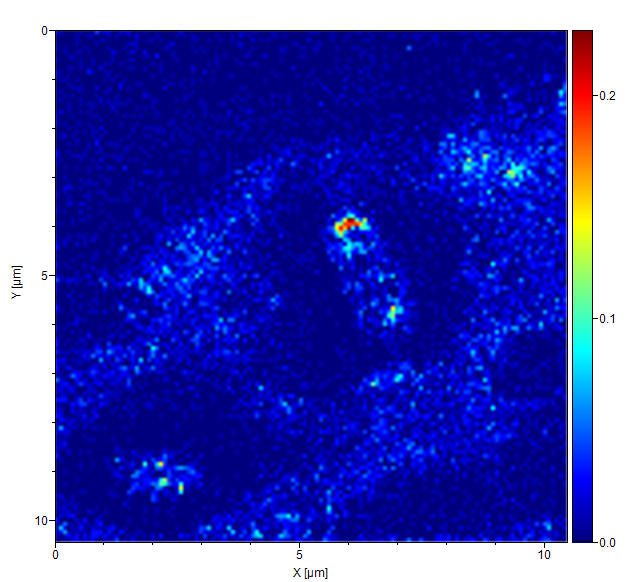

Supplement: Supplementary file 4 — Additional images from cryogenic FIB-SIMS imaging. [file 41564_2025_2032_MOESM4_ESM.zip › 20240507_Ecoli_PFNA_cell25.png]

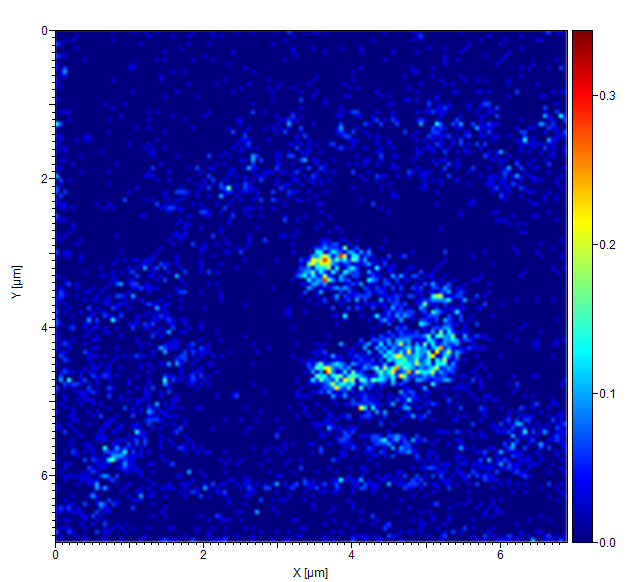

Supplement: Supplementary file 4 — Additional images from cryogenic FIB-SIMS imaging. [file 41564_2025_2032_MOESM4_ESM.zip › 20240507_Ecoli_PFNA_cell26.png]

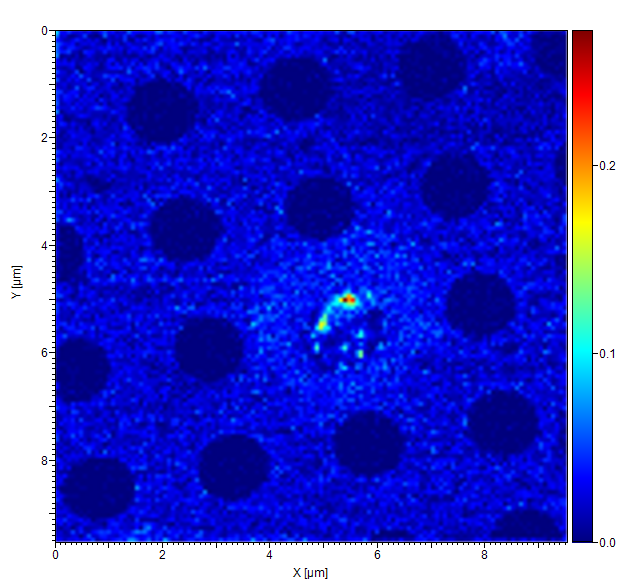

Supplement: Supplementary file 4 — Additional images from cryogenic FIB-SIMS imaging. [file 41564_2025_2032_MOESM4_ESM.zip › 20240507_Ecoli_PFNA_cell28.png]

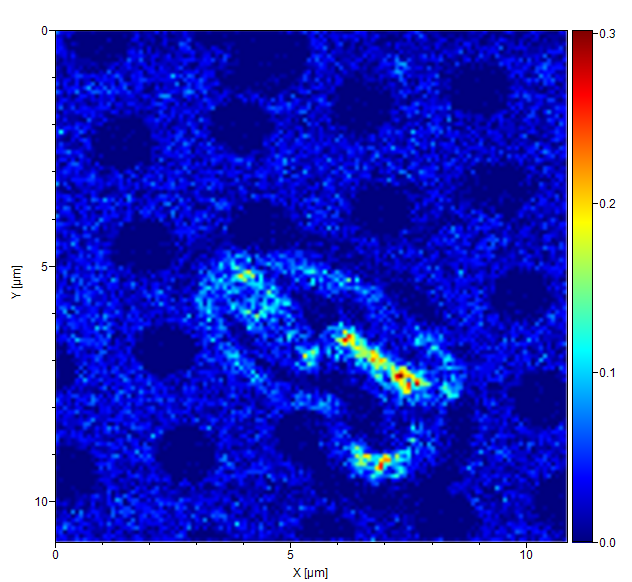

Supplement: Supplementary file 4 — Additional images from cryogenic FIB-SIMS imaging. [file 41564_2025_2032_MOESM4_ESM.zip › 20240507_Ecoli_PFNA_cell29.png]

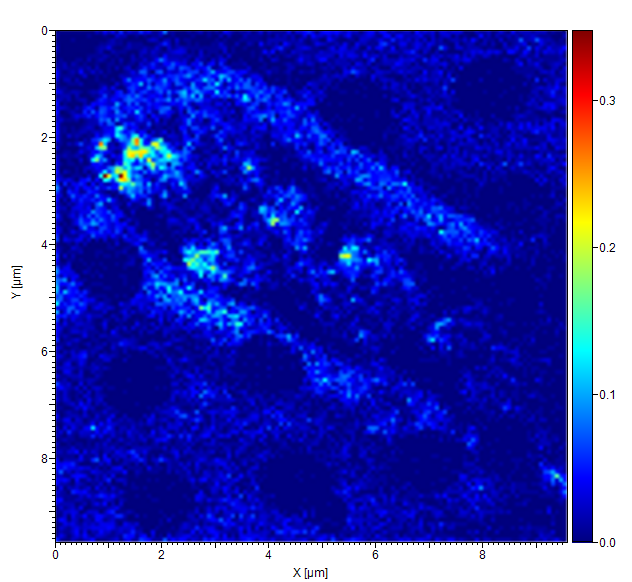

Supplement: Supplementary file 4 — Additional images from cryogenic FIB-SIMS imaging. [file 41564_2025_2032_MOESM4_ESM.zip › 20240507_Ecoli_PFNA_cell30.png]

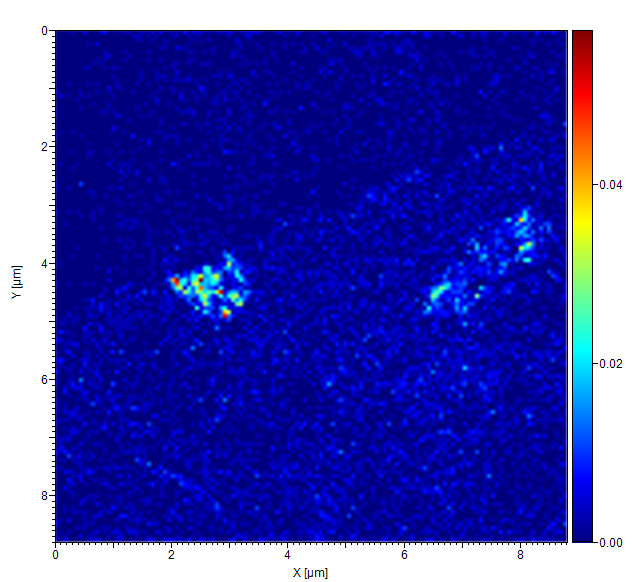

Supplement: Supplementary file 4 — Additional images from cryogenic FIB-SIMS imaging. [file 41564_2025_2032_MOESM4_ESM.zip › 20240507_Ecoli_PFNA_cell31.png]
